# A Data-Driven Approach for the Development of a Time-Informed Adverse Outcome Pathway Network for the Cardiotoxicity of Environmental Chemicals

**DOI:** 10.1021/acs.est.6c00396

**Published:** 2026-10-02

**Authors:** Alexandra Schaffert, Sivakumar Murugadoss, Tom Roos, Nunzia Linzalone, Gabriele Donzelli, Ronette Gehring, Birgit Mertens, Martin Paparella

**Affiliations:** † Mechanistic and Applied Toxicology (MAT), Faculty of Medicine and Health Technology, Tampere University, 33100 Tampere, Finland; ‡ Institute of Medical Biochemistry, Medical University Innsbruck, 6020 Innsbruck, Austria; § Tampere Institute for Advanced Study, Tampere University, 33100 Tampere, Finland; ∥ Health Effects Laboratory, Department of Environmental Chemistry and Health Effects, NILU The Climate and Environmental Research Institute, 2007 Kjeller, Norway; ⊥ Department of Population Health Sciences, Institute for Risk Assessment Sciences (IRAS), Faculty of Veterinary Medicine, Utrecht University, 3584 Utrecht, The Netherlands; # Institute of Clinical Physiology, National Research Council, 56124 Pisa, Italy; ¶ Scientific Department of Chemical and Physical Health Risks, 54513 Sciensano, 1050 Ixelles, Belgium

**Keywords:** AOP network, key event relationship, essentiality, temporality, oxidative stress, mitochondrial dysfunction, cardiac remodeling, non-animal methods

## Abstract

We developed a bottom-up, data-driven approach for constructing a time-informed adverse outcome pathway (AOP) network for environmental chemical-induced cardiotoxicity. Mechanistic end points were systematically extracted from 339 in vitro and in vivo studies, yielding 1759 Key Event (KE) entries and 4938 Key Event Relationship (KER) entries, together with study metadata and essentiality evidence from intervention experiments. After quality filtering and minimum support thresholding, 62 unique KEs and 256 unique KERs were retained for network construction. Network analysis highlighted oxidative stress and mitochondrial dysfunction as prominent, highly connected KEs linking diverse upstream perturbations to downstream cardiomyocyte injury, inflammation, cardiac remodeling (fibrosis and hypertrophy), decreased myocardial contractility, and reduced left ventricular function. A central methodological finding was that temporal information was more informative at the level of KERs than at the level of KEs. The same KE could occur across multiple durations depending on the relationship in which it was embedded, whereas KER-level time-scale annotation resolved distinct acute, subacute, and chronic pathway patterns not apparent from KE timing alone. This framework provides an evidence-weighted synthesis of the most consistently supported cardiotoxicity mechanisms and introduces a temporal layer for the mechanistic interpretation of heterogeneous evidence.

## Graphical abstract

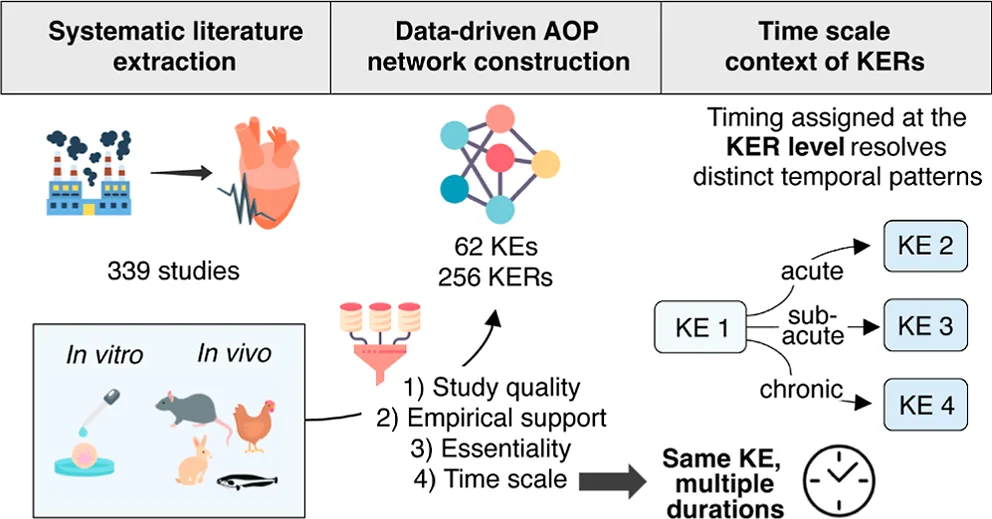

## Introduction

Cardiovascular diseases (CVDs) remain the leading cause of mortality globally, accounting for a substantial fraction of all deaths each year.^1^ Beyond established lifestyle and clinical risk factors, an expanding body of evidence indicates that environmental exposures, including industrial chemicals, complex mixtures, and ambient pollutants, contribute to cardiovascular morbidity.^2,3^ These exposures not only exacerbate pre-existing conditions like hypertension and heart failure but also contribute to the onset of cardiotoxicity.^4^ Despite this growing concern, substantial knowledge gaps remain in identifying the specific mechanisms through which environmental chemicals impact the cardiac function. These gaps present a significant barrier to the prediction and mitigation of chemically induced cardiotoxicity, emphasizing the urgent need for a robust framework to address these challenges.

Existing regulatory frameworks for chemical safety assessment insufficiently assess cardiotoxicity.^5^ For chemicals regulated under REACH, the Plant Protection Products Regulation, and the Biocidal Products Regulation, the evaluation of cardiotoxicity is not explicitly required. Instead, data on cardiotoxic effects are often derived indirectly from repeated-dose animal studies, which typically include end points such as cardiac weight, necropsy findings, and histopathology. While these studies provide some insight into potential cardiac toxicity, they do not capture detailed mechanistic information that is relevant to human health. Furthermore, the reliance on animal testing raises concerns about interspecies variability, resource demands, and alignment with the 3Rs (reduce, refine, and replace) principles. These challenges highlight the need for more targeted and mechanistically informed approaches to assessing cardiotoxicity in regulatory contexts.

Non-animal methods (NAMs), including human-induced pluripotent stem cell-derived cardiomyocytes (hiPSC-CMs), organotypic cardiac models, high-content phenotyping, and multi-omics, offer a route to generate human-relevant mechanistic signals for cardiotoxicity, with the potential to increase throughput and improve biological interpretability. However, NAM data cannot be used optimally in isolation: regulatory application requires structured approaches that integrate diverse mechanistic and apical evidence into coherent causal narratives that support hazard identification, weight of evidence evaluation, and context of use decisions. Reflecting this need, cardiotoxicity is increasingly discussed as an emerging target for harmonization and methodological development in international hazard assessment, including an OECD Working Party on Hazard Assessment (WPHA) initiative addressing cardiotoxicity as a potential hazard class and the role of novel methods in its evaluation.^6,7^

The Adverse Outcome Pathway (AOP) framework offers a structured approach to connect molecular-level perturbations caused by chemical exposures to higher-order adverse outcomes (AOs), such as heart failure or reduced cardiac function.^8^ AOPs rely on key events (KEs) and key event relationships (KERs) to systematically link mechanistic data to observable AOs, thereby providing a transparent basis for mechanistically informed risk assessment. By integration of data generated with NAMs into AOPs, pathways leading to cardiotoxicity can be elucidated and critical points of intervention or regulatory relevance identified. Importantly, cardiotoxicity is unlikely to be captured by single linear pathways; rather, multiple converging and diverging processes can culminate in shared cardiac outcomes. AOP networks therefore provide an appropriate representation of mechanistic complexity and enable the identification of high-confidence, recurrent pathways across stressors and evidence streams.^9^ Despite significant progress in AOP development for other toxicological end points, applications targeting cardiotoxicity remain scattered and often focus on individual pathways or predefined mechanisms, leaving a substantial gap in systematic, human-relevant AOP coverage for cardiac effects of environmental pollutants.

Here, we address this gap by using a bottom-up, data-driven strategy to develop an AOP network for environmentally chemical-induced cardiotoxicity. Building on our prior systematic evidence map (SEM),^10,11^ we harmonized mechanistic observations from in vitro and in vivo studies into candidate KEs and KERs and constructed a network from recurring literature patterns rather than predefined pathways. Adding literature-derived time scale information at the KER level provides temporal context for pathways linking early perturbations to adverse cardiac outcomes.

## Methods

### AOP Development Strategy Overview

The workflow used to develop the cardiotoxicity AOP network is shown in Figure 1. The strategy was informed by the AOP Developers’ Handbook, particularly its recommendations to document the evidence-identification strategy, the rationale for KE and KER selection, and the evidence dimensions used to evaluate KER confidence, including biological plausibility, empirical support, essentiality, and quantitative or time-scale understanding. In contrast to a formal OECD endorsement-level AOP assessment, the aim of the present study was to develop a transparent, literature-derived, bottom-up AOP network for environmental chemical-induced cardiotoxicity and to annotate this network with evidence-weighting and time-scale information.

**Figure 1:**
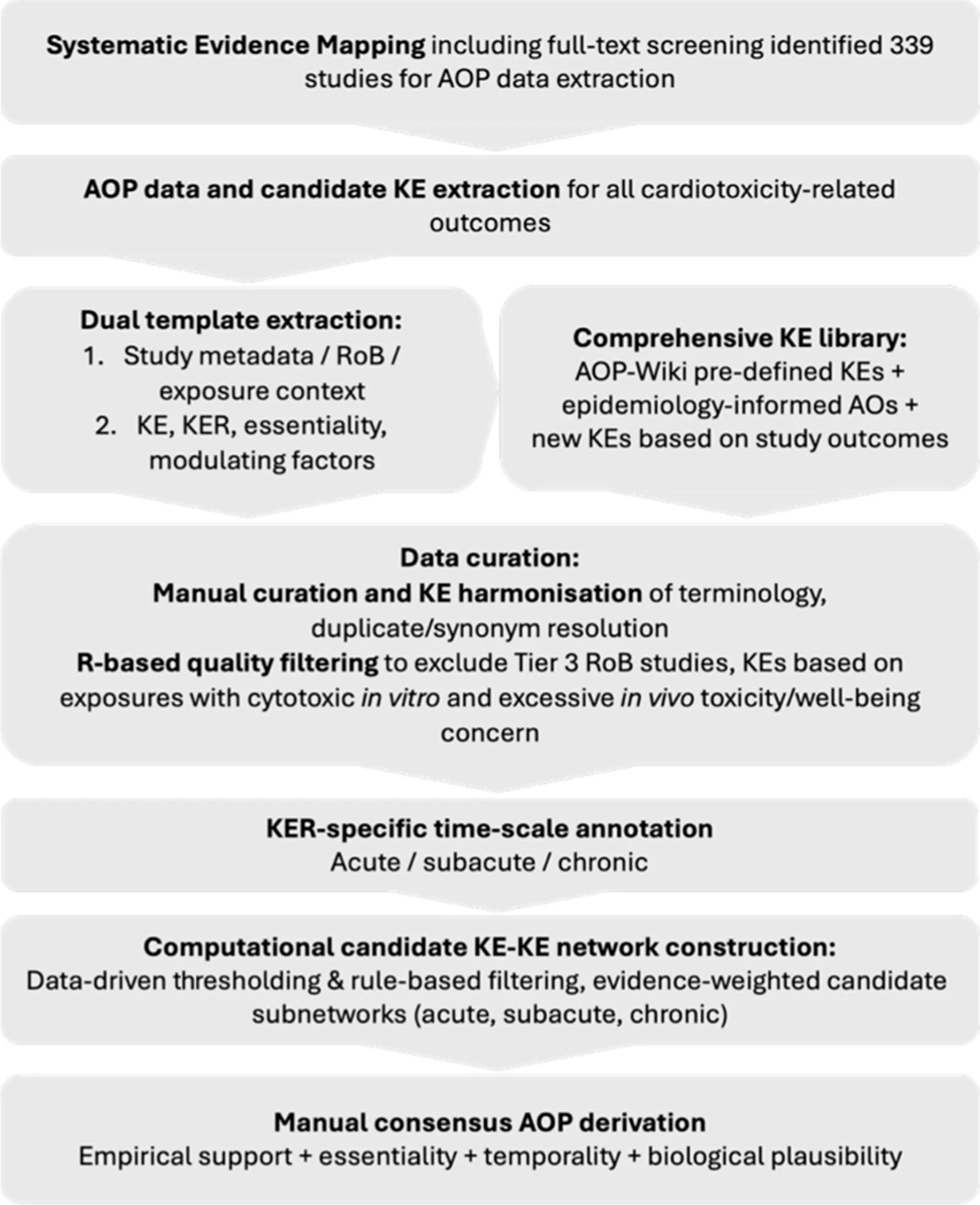
Data-driven and evidence-weighted workflow for cardiotoxicity AOP network development. Starting from a systematic evidence map, studies with at least two potential KEs were selected for AOP-relevant extraction. Extracted KE/KER information was harmonized, quality-filtered, and processed in R to generate a data-derived candidate KER network. Candidate KERs were retained based on minimum independent replication, refined using a predefined biological-plausibility rule, and annotated with KER-specific time scales. Acute, subacute, and chronic evidence-weighted candidate subnetworks were then manually consolidated into a final consensus AOP network using empirical support, essentiality-supported directionality, biological plausibility, and temporal context.

The workflow consisted of four main stages: (i) systematic evidence mapping and AOP-relevant data extraction; (ii) KE harmonization, quality filtering, and candidate KER construction; (iii) rule-based biological plausibility refinement and evidence weighting; and (iv) KER-specific time-scale annotation and consensus network development. The initial network construction was data-driven and implemented computationally, whereas biological plausibility refinement combined predefined structural rules with an expert review against AOP weight-of-evidence principles.

### AOP Data Extraction and KE Vocabulary Harmonization

Literature data for the development of a cardiotoxicity AOP network was extracted from a systematic evidence map (SEM) of studies related to environmental pollutant exposure leading to cardiotoxicity in vitro and in vivo, as previously described.^10,11^ The SEM was designed around outcomes associated with or capable of contributing to contractile dysfunction as a proxy for heart failure. Accordingly, the resulting AOP network primarily represents mechanisms connected to myocardial injury, structural remodeling, and functional impairment and may provide less complete coverage of cardiac effects not linked to contractile dysfunction within the included studies. End points that had previously been identified in our epidemiological review of cardiotoxic effects associated with environmental chemical exposures in humans^12^ were considered as potential AOs, and thus, terminology and definition criteria were harmonized to match those used in the epidemiological study. This ensured consistency in the annotation and human relevance of these outcomes within the AOP networks.

Of the 400 studies included in the final systematic evidence mapping, 339 contained mechanistic information on at least two potential KEs and were therefore eligible for AOP-relevant data extraction, while the remaining 61 studies were excluded because of insufficient outcome coverage. Relevant data were extracted from two initial templates. The first template included general study information, which encompassed details of study design, experimental setup, and a risk of bias (RoB) assessment conducted according to OHAT criteria (Table S1). The RoB assessment categorized studies into three tiers based on 11 questions, including two key questions, and was evaluated with a scoring system.^10,11^ The second template focused on AOP-relevant data, specifically targeting evidence to support the development of a cardiotoxicity AOP network (Table S1). Further details on data extraction and KE harmonization can be found in Supporting Information 1.

### Data Curation, Quality Filtering, and Processing

Following data extraction, all entries underwent manual curation to ensure consistency in KE assignments and correct any extraction-related errors. Subsequent data processing and candidate KE–KE network construction were performed in R Studio (version 4.4.1).

Quality filters were applied before the network construction. Entries were excluded if they originated from tier 3 studies based on RoB assessment, in vitro studies associated with cytotoxic concentrations (as assessed within the respective studies), and in vivo studies reporting excessive body weight loss or mortality. Closely related KEs were also consolidated, where appropriate, to improve interpretability and avoid unnecessary fragmentation. For example, multiple signaling events associated with cardiac hypertrophy or fibrosis were grouped under unified KE descriptors when they represented shared biological functions.

All extracted and curated AOP-relevant data generated during this study are compiled in Supporting Information 2.

### KER Time Annotation

To characterize the temporal progression of cardiotoxic effects, we first assigned an exposure duration to each KE-study record based on the shortest duration at which a statistically significant modulation of that KE was reported. If a study reported only one time point, then that duration was used. If multiple time points were assessed, we extracted the earliest time point at which the KE showed a statistically significant change compared to the control (as defined by the study authors). Free-text descriptions of exposure schedules were parsed and converted to numeric exposure durations in days. Because the data set covered multiple species and test systems with different typical study durations, we harmonized exposure times using species- and system-specific cut-offs anchored in OECD Test Guidelines and common toxicology study designs. These cut-offs were defined a priori for each experiment type (in vivo vs in vitro) and for each species category (mice, rats, zebrafish, Drosophila, swine, chickens, rabbits, quails, tadpoles, marmosets, and in vitro systems). Exact thresholds and supporting references are summarized in Supporting Information 1, Table S1. Each KE-study record was then assigned to one of four initial duration categories: acute, subacute, subchronic, or chronic. For descriptive summaries and network visualization, subchronic and chronic categories were combined into a single chronic class because explicitly chronic studies were overall rare (Supporting Information 1, Figure S6) and both subchronic and chronic designs represent sustained perturbations compared with shorter-term exposures. The full category classification and all species-specific thresholds are documented in Supporting Information 1.

All duration categories were assigned from the reported exposure times by using species-specific time windows derived from typical test guideline durations and used as heuristic anchors. Exposure durations were not adjusted for species differences in the internal dose or accumulation. Thus, the categories represent a structured grouping of study designs rather than harmonized internal exposure conditions.

### Evidence-Weighted AOP Network Construction

Empirical support was operationalized at the network level as recurrence across independent observations, essentiality was assessed where intervention evidence was available, biological plausibility was considered through predefined structural rules and expert review, and KER-specific time-scale annotation was added.

Candidate KERs were generated computationally in R Studio (version 4.4.1) from KE pairs observed within the same experimental context, defined as a distinct in vivo or in vitro experiment. These candidate linkages were not interpreted as being causal by default. At the network level, empirical support was operationalized as recurrence of the same candidate KER across independent observations, reflecting the replication/consistency aspect of AOP weight-of-evidence reasoning. Candidate KERs meeting the minimum replication threshold of at least three independent study observations were further evaluated. Sensitivity analyses across the alternative thresholds were performed.

Dose–response concordance is a recognized element of AOP empirical support, but due to substantial heterogeneity in chemical identity, experimental design, and dose selection across the included studies, dose–response information could not be meaningfully harmonized; dose concordance analysis was therefore not performed. This mainly affects interpretation of early stress-response and functional KEs (including decreased contractility), where adaptive versus adverse transitions may be dose-dependent, whereas later injury/remodeling KEs are less ambiguous once observed.

Rule-based biological plausibility refinement was applied after the candidate KER generation. Each KE was manually annotated with a biological level of organization. A predefined structural rule was then applied in R to exclude candidate KERs that directly linked molecular-level KEs to organ- or organism-level KEs when intermediate cellular- or tissue-level events were bypassed. Such links were considered mechanistically noninterpretable because they skipped intermediate biological levels required for a plausible event sequence.

The retained candidate KERs were stratified by the KER-specific exposure-duration category to generate acute, subacute, and chronic evidence-weighted candidate subnetworks. To provide an objective representation of the strongest paths within the candidate subnetworks, evidence-prioritized paths were identified separately for acute, subacute, and chronic time scales. KER scores combined log-transformed empirical recurrence and direction-specific essentiality support, with greater weight being assigned to essentiality. Essentiality-supported directions were retained irrespective of the biological level, whereas empirical-only KERs were oriented according to progression across biological levels. Paths were ranked using the geometric mean of their constituent KER scores, thereby accounting for the evidence strength across the complete path without automatically favoring longer paths. The top 5 ranked paths were highlighted for an exploratory investigation. The final consensus network was manually consolidated from the complete candidate subnetworks and was not derived solely from the top paths. This expert-based consolidation considered empirical support, essentiality-supported directionality, KER-specific temporality, and biological plausibility. Further details can be found in Supporting Information 1. The final product is a consensus, time-resolved cardiotoxicity AOP network that captures the most empirically supported and temporally coherent pathways emerging from an evidence map.

## Results

Across the extracted data set, in vivo evidence contributed the majority of KE entries, with in vitro studies representing a smaller but methodologically diverse fraction (Supporting Information 1, Figure S1A). Within the in vivo evidence stream, rodent models dominated, with rats most frequently represented followed by mice; all other species (e.g., chicken, quails, zebrafish, swine/pigs, rabbits, Drosophila, tadpoles, and marmosets) contributed comparatively fewer KE entries (Supporting Information 1, Figure S1B). In vitro KE entries were distributed across a broad set of cardiac-relevant cellular systems, led by primary cardiomyocytes, H9c2 cells, and human cardiomyocytes, with additional coverage from endothelial and fibroblast models (e.g., EA.hy926, HUVEC, neonatal cardiac fibroblasts) and stem cell-derived platforms (e.g., hiPSC- and mESC-derived cardiomyocytes) (Supporting Information 1, Figure S1C). Collectively, this model landscape indicates that the mechanistic evidence base used here is anchored in mammalian in vivo biology, while simultaneously capturing human-relevant cellular models that are directly compatible with NAM-oriented mechanistic testing and provide coverage for multi-cell-type processes (e.g., vascular/endothelial contribution and fibrosis-related mechanisms).

To make the contribution of different model systems explicit, we summarized the distribution of evidence across taxa and experimental systems for the main network relationships (top 40 KERs by support; Supporting Information 1, Figure S2). The evidence base is heavily rodent-dominated (Supporting Information 1, Figure S1B) with rat and mouse studies accounting for most observations and covering nearly all highly supported KERs, which reflects the disproportionate use of these species in the cardiotoxicity literature. Within this distribution, rat evidence contributes most strongly to KERs centered on oxidative stress associations, while mouse evidence appears to disproportionately cover relationships closer to functional/adverse outcomes, including KERs involving decreased LV function and decreased myocardial contractility. In vitro evidence contributes disproportionately to molecular and cellular mechanisms, particularly mitochondrial/energy-related linkages (e.g., decreased ATP production and mitochondrial dysfunction), consistent with the mechanistic resolution of cellular systems. Resolving whether the observed trends reflect true translational differences will require targeted cross-species comparative studies measuring the same KE/KER linkages under matched experimental designs.^13^

From 339 studies included in the extraction, 1759 KE entries and 4938 KER entries were retrieved (Figure 2). After quality filtering (high risk of bias, confounding cytotoxicity in vitro, excessive toxicity or animal well-being concerns in vivo), 112 unique KEs and 869 unique KERs were retained. The AOP framework is intended to support credible, transparent hazard assessment reasoning, and OECD AOP guidance explicitly operationalizes confidence using tailored Bradford-Hill considerations, where empirical support includes incidence/replication, i.e., the extent to which relationships are supported across independent observations and interventions. Thus, to focus the network on well-replicated mechanistic relationships and reduce the influence of single-source findings, we applied an additional inclusion threshold of ≥3 independent observations per KER, yielding a final network of the most robust 62 KEs and 256 KERs used for downstream analyses. The robustness of this threshold choice was confirmed by sensitivity analysis across thresholds from ≥1 to ≥6 and reflects a pragmatic balance between increasing confidence in replicated relationships and retaining sufficient coverage for network-level interpretation (Supporting Information 1, Figures S3 and S4, and Table S2).

**Figure 2:**
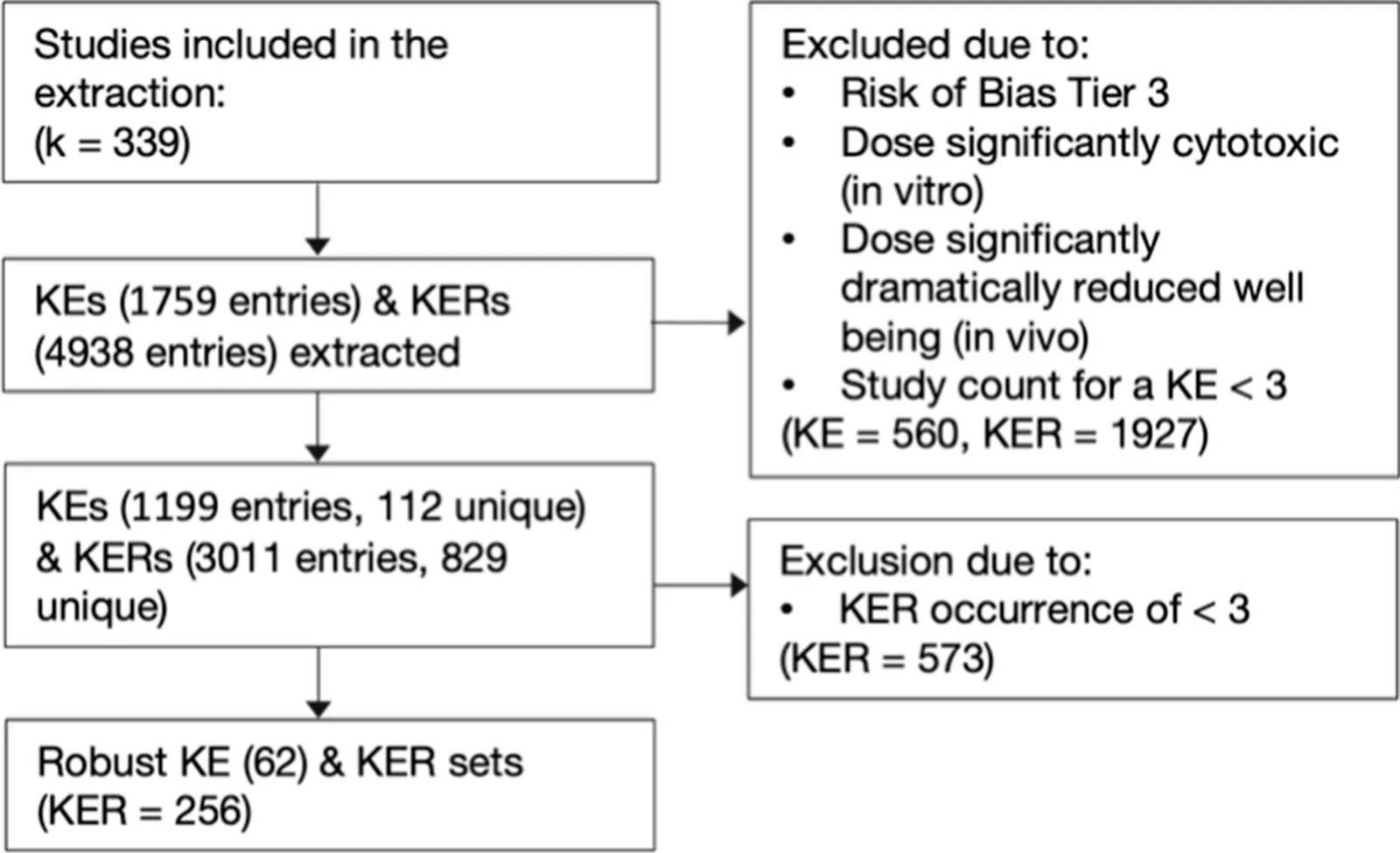
Flowchart of filtering process of literature extracted AOP data.

To prioritize mechanistically substantiated relationships and support directionality, we constructed an essentiality-informed network based on intervention evidence. Essentiality-supported KERs were defined as relationships in which targeted perturbation of an upstream KE (e.g., pharmacological inhibition or genetic manipulation) resulted in a measurable change in a downstream KE (Figure 3). Among the total 829 unique KERs, 109 (13.1%) are supported within this essentiality-supported subset, *Increase oxidative stress* appeared most frequently. Specifically, essentiality-supported links were most often reported between *Increase oxidative stress* and downstream cardiotoxicity-relevant events such as *Increase cardiomyocyte cell death* (36 relationships), *Inflammation* (*n* = 15), *Increase mitochondrial dysfunction* (*n* = 8), and *Increase pro-hypertrophic signaling* (*n* = 8) and the remodeling events *Cardiac fibrosis* (*n* = 4) and *Cardiac hypertrophy* (*n* = 3). While oxidative stress was the most interconnected node, bidirectional interactions were also observed. For example, *decreased antioxidant capacity* emerged both as a downstream consequence (*n* = 26) and as an upstream driver of oxidative stress. Similarly, *mitochondrial dysfunction* and *inflammation* were reciprocally linked to oxidative stress, consistent with feedback interactions described in the cardiovascular literature.^14,15^ The presence of aryl hydrocarbon receptor (AhR) activation within the network, although with fewer essentiality-supported relationships (*n* = 2), suggests that this MIE may act through modulation of oxidative stress and its downstream consequences.

**Figure 3:**
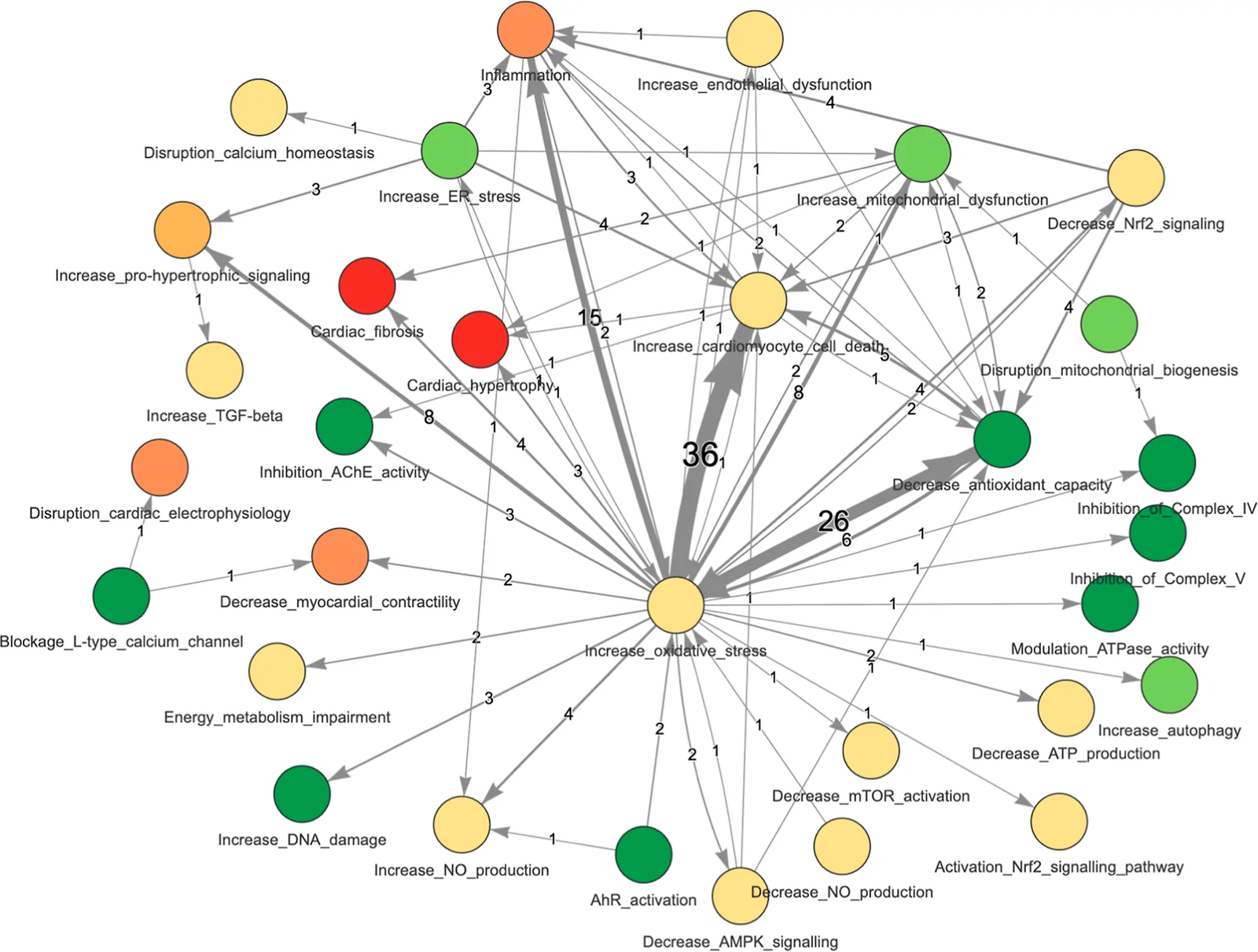
Network of extracted essentiality relationships. Edges represented essentiality relationships, with their thickness proportional to the essentiality count. Nodes represent KEs and are color-coded based on their biological level (dark green: molecular, light green: subcellular; yellow: cellular; light orange: multicellular; orange: tissue; red: organ/organism).

To contextualize thte network structure in time, we assessed whether exposure-duration categories exhibited a consistent monotonic pattern across biological levels (MIE → intermediate KEs → AOs). No generalizable trend was observed at the level of biological level categories (Supporting Information 1, Figure S5), indicating that “level” alone does not reliably encode temporal progression in the extracted evidence base. We found that for each KE, observations are distributed across acute, subacute, and chronic time scales depending on which specific KER it participates in, as exemplified by the hub KE *increase oxidative stress* (Figure 4A). In other words, the same KE does not have a single characteristic “time”, but its temporal context shifts with the relationship in which it is embedded. We therefore evaluated temporality at the KER level. Accordingly, we assigned each KER the most frequently observed exposure-duration category of both connected KEs, yielding a KER-specific temporal scale annotation that can be propagated across the network to visualize time-resolved mechanistic paths (Figure 4B–D). Of note, because the temporal categories here reflect reported exposure durations, cross-system comparisons (e.g., ‘subacute’ in vivo vs ‘subacute’ in vitro), our KER-level temporality is intended to support within-evidence-base ordering and time scale annotation, not cross-species kinetic inference. Within each temporal subnetwork, the five highest-ranked evidence-prioritized paths were additionally highlighted to identify the strongest and most consistently supported mechanisms.

**Figure 4:**
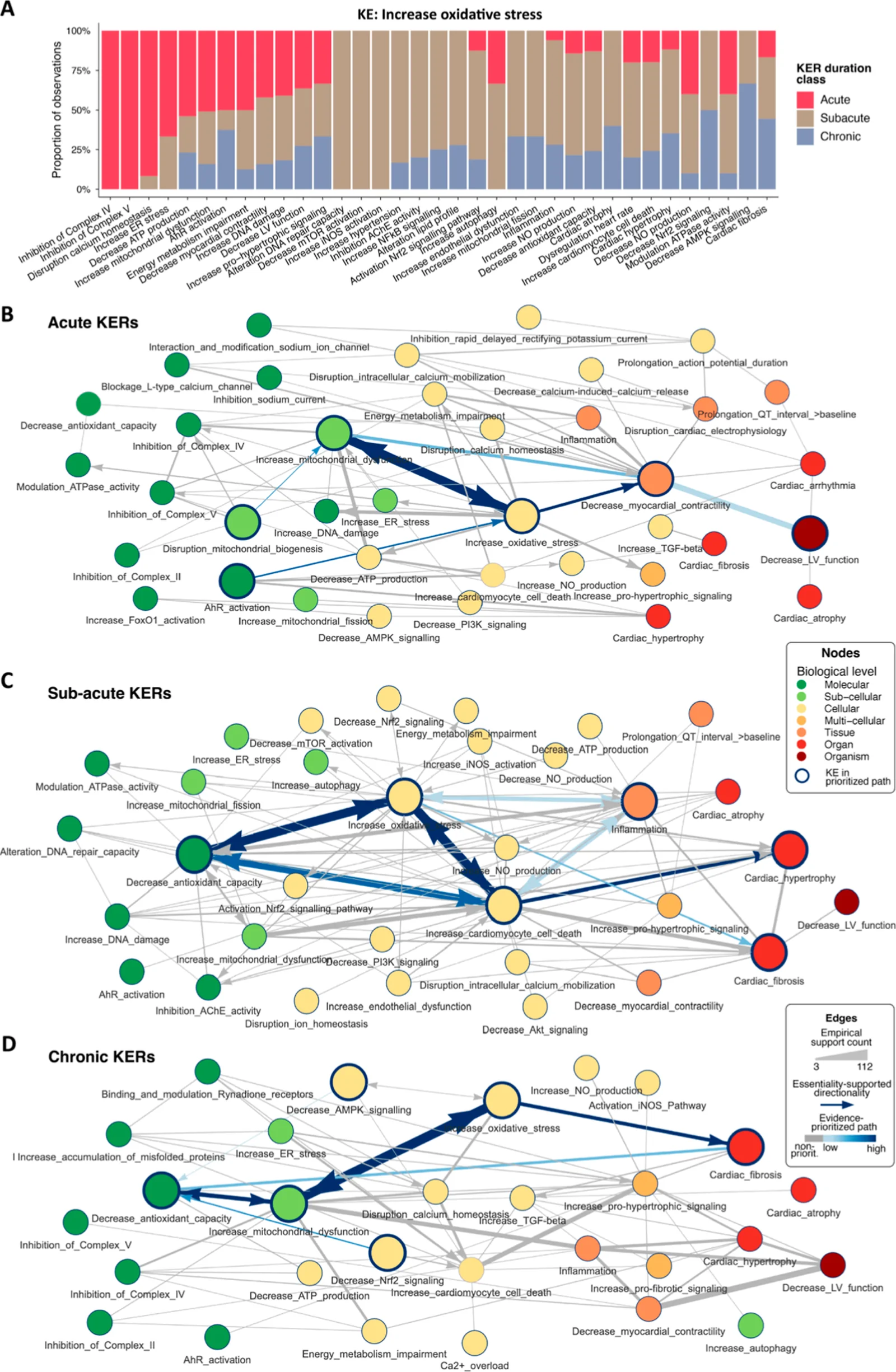
Duration-organized, evidence-weighted cardiotoxicity adverse outcome pathway (AOP) subnetworks and Key Event Relationship (KER)-specific temporal scale for a representative Key Event (KE). (A) Example KE “*Increase oxidative stress*” showing for connected KE observed within the same experiment (x-axis), the proportion of observations assigned to acute, subacute, or chronic exposure-duration classes (y-axis), illustrating that the same KE can occur across different exposure windows depending on the specific upstream/downstream relationship. (B–D) Subnetworks of KERs assigned to (B) acute, (C) subacute, and (D) chronic duration classes. Nodes represent KEs and are color-coded by biological level (legend). Edge thickness is proportional to empirical support (KER count). Arrows on the edges indicate essentiality-supported directional KERs. Edge darkness indicates membership in and overlap among the top five evidence-prioritized paths, edge width represents empirical support, arrows indicate essentiality-supported directionality, and blue node borders identify KEs included in at least one prioritized path. Top-ranked paths were identified using combined empirical and essentiality support.

Notably, in vitro-derived KE entries were predominantly acute (Supporting Information 1, Figure S6), reflecting typical short in vitro exposure windows; longer in vitro designs occurred but were uncommon, and this bias is unlikely to affect overall patterns because in vitro entries represent a minority of the data set (Supporting Information 1, Figure S1). Interactive visualizations of all KE networks are provided as Supporting Information 3.

### Acute Subnetwork: Early Functional Perturbation Converging on Contractility and Electrophysiology

In the acute subnetwork (Figure 4B), electrophysiology- and calcium-handling-related KERs are present but are generally sparse and weakly supported. These include ion channel and action potential-related events that empirically link to *disruption of cardiac electrophysiology*, *prolongation of action potential* or QT interval, and *cardiac arrhythmia*. For a subset of these relationships, including *L-type calcium channel blockade* and *increased late sodium current*, directionality is additionally supported by essentiality evidence. Although these electrophysiological relationships remained peripheral rather than dominant network hubs, they connect to *decreased myocardial contractility*, placing acute electrical disruption in proximity to early functional impairment.

Molecular and subcellular perturbations are also evident in the acute window. Inhibition of mitochondrial respiratory chain complexes and *AhR activation* is connected to intermediate cellular stress responses, including *increased oxidative stress*, *increased mitochondrial dysfunction*, *decreased ATP production*, and energy metabolism impairment, with intervention-based essentiality support specifically for the *AhR activation* to *oxidative stress* linkage. These stress- and energy-related KEs are more densely connected than electrophysiological events in the acute network and frequently converge on *decreased myocardial contractility*; among these, selected links, including *oxidative stress* to *decreased myocardial contractility*, are supported by essentiality evidence. Together, the acute subnetwork indicates that early cardiotoxic effects are driven primarily by rapid metabolic and stress-related perturbations with electrophysiological alterations appearing as additional but less consistently supported events. Accordingly, the evidence-prioritized paths converge predominantly through *mitochondrial dysfunction* and *oxidative stress* on *decreased myocardial contractility*, with *AhR activation* and *disrupted mitochondrial biogenesis* appearing as upstream events and one prioritized path extending to *decreased LV function*.

### Subacute Subnetwork: Shift toward Injury, Antioxidant Capacity, and Inflammation

In the subacute subnetwork (Figure 4C), the network structure shifts toward cellular injury and inflammatory processes as dominant organizing features. *Increase cardiomyocyte cell death* emerges as a central hub with strongly connected upstream KEs, including *increase oxidative stress*, *decrease antioxidant capacity*, and *increase mitochondrial dysfunction*. Notably, all three show essentiality-supported directional links to increased cardiomyocyte cell death. Downstream, *increased cardiomyocyte cell death* links to tissue- and organ-level outcomes, including *cardiac fibrosis* and *cardiac hypertrophy*; among these, the cell death to hypertrophy relationship is additionally supported by essentiality evidence. *Inflammation* becomes a second highly connected node in the subacute network. Its positioning downstream of oxidative stress and cardiomyocyte injury is supported by essentiality evidence, whereas its onward connections to fibrotic and hypertrophic events are supported by empirical recurrence in the network. Accordingly, the five evidence-prioritized paths linked *decreased antioxidant capacity* and *oxidative stress* to *cardiomyocyte cell death* and *cardiac hypertrophy* or *fibrosis*, with *inflammation* incorporated as an intermediate event in one of the prioritized paths.

Compared with the acute subnetwork, the subacute network contains a richer set of intermediate regulatory KEs, including mitochondrial dynamics (e.g., mitochondrial fission), stress-response signaling (e.g., Nrf2-related changes), and homeostatic processes such as autophagy and mTOR signaling. Overall, the subacute subnetwork marks a transition from early functional disturbance to accumulating cellular damage and inflammatory signaling that mechanistically links initial stress responses to emerging structural remodeling.

### Chronic Subnetwork: Sustained Stress-Mitochondrial Dysfunction Coupled to Remodeling and LV Function Decrease

In the chronic subnetwork (Figure 4D), the network structure becomes more compact and is dominated by strongly evidence- and essentiality-supported KERs for *increase oxidative stress*, *decrease antioxidant capacity*, and *increase mitochondrial dysfunction* with extensive links to inflammatory and remodeling-related processes. *Cardiac fibrosis* emerges as a key tissue-level outcome in the chronic network and as a recurrent end point of the highest-ranked evidence-prioritized paths, with an essentiality-supported directional link from *oxidative stress* and additional empirical connection to the pro-fibrotic KE *increased TGF-beta*. *Cardiac hypertrophy* is placed alongside *oxidative stress* and *prohypertrophic signaling* and supported in part by essentiality evidence, whereas its broader links to inflammatory and functional impairment KEs are supported by empirical recurrence in the network. *Decrease myocardial contractility* and *decrease LV function* are now tightly embedded within the chronic network, connecting to sustained stress, mitochondrial dysfunction, inflammation, and structural remodeling KEs; among these, selected upstream links from oxidative stress and mitochondrial dysfunction are supported by essentiality evidence. Calcium-related disturbances and *increase ER stress* primarily connect through injury-related end points such as *cardiomyocyte cell death* rather than acting as dominant organizing features. Collectively, the chronic subnetwork indicates that long-term cardiotoxicity is marked by persistent oxidative and mitochondrial stress, leading to inflammatory amplification, structural remodeling, and progressive decline in ventricular function.

### Modulating Factors and Potential Prototypical Stressors

Several modulating factors were reported to influence the occurrence or magnitude of cardiotoxic KEs (Figure 5A). A high-fat diet was the most frequently reported modifier and was associated with amplification of structural, metabolic, and inflammatory KEs, including *cardiac hypertrophy*, *energy metabolism impairment*, and *inflammation*. Pre-existing cardiovascular conditions similarly intensified downstream KEs, particularly *increased oxidative stress*, cell injury/death, and *cardiac fibrosis*. Sex-specific effects were observed in a small number of studies, mainly affecting cell injury/death and cardiac remodeling KEs, but the limited evidence precludes generalization. Overall, modulating factor information was inconsistently reported, highlighting substantial gaps in contextual data relevant to cardiotoxicity susceptibility.

**Figure 5:**
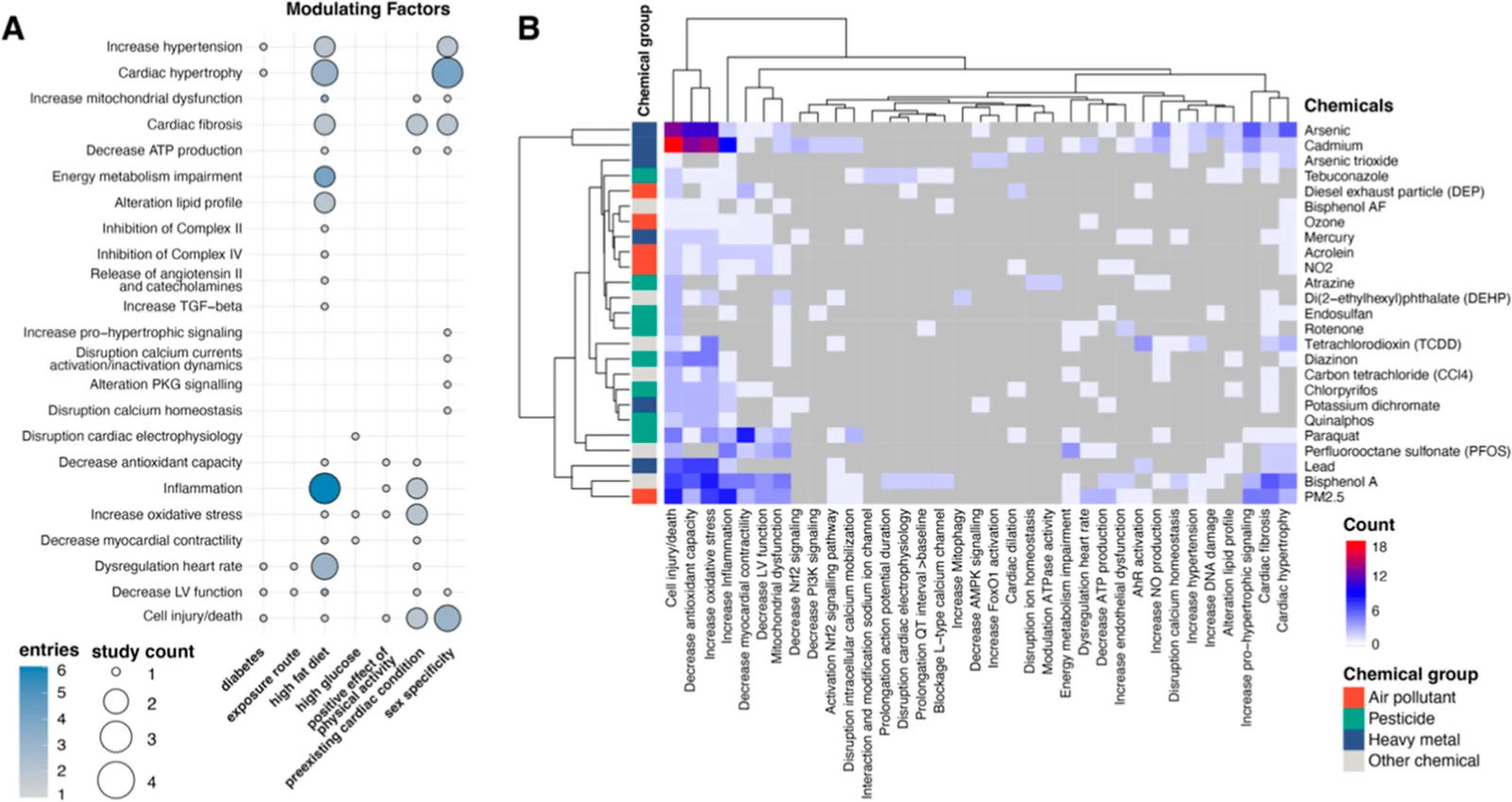
Modulating factors and chemical-KE associations. (A) Bubble plot showing seven modulating-factor categories that influenced cardiotoxic KEs. (B) Heat-map of chemicals with top associations, based on chemicals found with at least 10 associations, and KEs with at least 4 associations. Chemical group and number of studies that investigated the chemicals are indicated on the right side.

To explore whether specific environmental chemicals preferentially associate with particular KEs in the cardiotoxicity AOP network, we analyzed stressor-KE co-occurrence patterns for chemicals with sufficient representation in the data set (Figure 5B). Across chemicals, *increase oxidative stress, inflammation, and increase mitochondrial dysfunction* emerged as the most frequently reported KEs, consistent with their central positioning in network analysis. Several stressors showed recurrent associations with these pathways rather than with highly specific downstream outcomes. In particular, the heavy metals cadmium and arsenic were strongly associated with the MIE *decreased antioxidant capacity* and with downstream KEs including *increased oxidative stress* and *cell injury/death*. Lead exhibited a similar association pattern, albeit supported by fewer studies, resulting in a lower overall evidence density. Cadmium additionally showed a comparatively strong association with *inflammation*, reinforcing its link to stress- and injury-driven cardiotoxic mechanisms. The persistent organic pollutant 2,3,7,8-tetrachlorodibenzo-p-dioxin (TCDD) was frequently associated with *AhR activation* and *increase oxidative stress*, reflecting its well-characterized mode of action and supporting the inclusion of *AhR activation* as a molecular-level entry point into the cardiotoxicity network. To evaluate whether the lack of clear clustering reflects true mechanistic similarity versus evidence-structure constraints, we additionally examined row-normalized chemical-KE profiles (controlling for uneven study coverage) and tested for KE enrichment using FDR correction (Figure S7). This analysis confirms broad convergence on shared stress/injury processes across many stressors, consistent with the stressor-agnostic core network while also revealing a limited set of statistically supported stressor-specific tendencies. For example, atrazine shows enrichment for *mitochondrial dysfunction* and *disruption of ion homeostasis*, TCDD for *AhR activation*, paraquat for *decreased myocardial contractility*, and DEHP for *increased mitophagy* (Supporting Information 1, Figure S7). At the chemical-class level, heavy metals show enrichment for *decreased antioxidant capacity* and *decreased myocardial contractility*, whereas air pollutants show enrichment for *decreased myocardial contractility* and *decreased LV function* (Supporting Information 1, Figure S8). As a result, the extracted evidence preferentially supports mechanistic convergence on stressor-agnostic, shared stress, and injury pathways, rather than stressor-specific, KE-distinct cardiotoxic signatures. A full list of chemical-KE associations is available in Supporting Information 2.

## Discussion

Despite increasing recognition of environmental contributors to CVD, cardiotoxicity remains poorly represented in current AOP resources and is rarely addressed through systematic pathway-resolved hazard frameworks. Here, we address this gap by applying a strictly bottom-up, literature-driven AOP development strategy to derive a mechanistically structured and time-resolved cardiotoxicity AOP network for environmental chemicals. By coupling systematic evidence mapping^16,17^ with evidence-weighted network assembly and KER-level temporal annotation, we provide a high-confidence synthesis of the cardiotoxicity mechanisms most consistently supported across heterogeneous in vitro and in vivo studies, together with an interpretable temporal context for sequence-of-event reasoning.

Our approach is grounded in evidence extracted from 339 studies identified via a systematic evidence mapping of pollutant-induced cardiotoxicity. After quality filtering, the final network construction was based on 62 harmonized KEs and 256 unique KERs supported by at least three independent observations. Directionality was assigned where essentiality evidence was available (i.e., targeted perturbation of an upstream KE altered a downstream KE), and exposure-time information was harmonized across species and test systems to enable categorization of KERs into acute, subacute, and chronic windows. The resulting network integrates three complementary evidence layers: empirical support, mechanistic directionality (where available), and temporal context.

### Mitochondrial and Redox Perturbations Form the Central Cardiotoxicity Network

Data-driven assessment of the KER frequency and essentiality highlighted oxidative stress and mitochondrial dysfunction as the most consistently represented events in the cardiotoxicity network. This is biologically plausible given the high metabolic demand of cardiac tissue and the established roles of redox imbalance and mitochondrial dysfunction in cardiomyocyte injury, contractile impairment, and remodeling. At the same time, oxidative stress, mitochondrial dysfunction, and inflammation are commonly measured end points in toxicology, and their prominence may therefore reflect a combination of biological relevance, assay availability, end point popularity, and publication/reporting practices.

The cardiac relevance of these stress-response KEs is nevertheless well supported. Oxidative stress contributes to cardiac remodeling and heart failure progression through effects on excitation–contraction coupling, hypertrophic signaling, apoptosis, and extracellular matrix remodeling. Similarly, mitochondrial dysfunction is particularly consequential in cardiomyocytes because of their high mitochondrial density, continuous ATP demand, and tight coupling between mitochondrial metabolism, calcium handling, and contractile function. Mitochondrial perturbation can therefore propagate energetic deficit, redox imbalance, disrupted Ca^2+^ handling, inflammatory amplification, and impaired contractility, providing a plausible mechanistic link between mitochondrial/redox stress and downstream cardiac remodeling or ventricular dysfunction.^14,15,18,19^

Our quality-filtering strategy reduces the likelihood that the network is dominated by nonspecific terminal toxicity: tier 3 studies, in vitro observations at cytotoxic concentrations, and in vivo observations under excessive toxicity or poor animal well-being were excluded. However, such filtering cannot fully remove bias arising from end point popularity or publication/reporting practices. Accordingly, the centrality of oxidative stress, mitochondrial dysfunction, and inflammation should be interpreted as reflecting a combination of biological relevance and the evidence availability.

Their cardiotoxic relevance in this network derives from their KER context rather than from their occurrence as isolated stress-response end points.^20^ Oxidative stress and mitochondrial dysfunction repeatedly connect to cardiac-relevant downstream KEs/AOs, including cardiomyocyte injury/death, impaired contractility, cardiac remodeling, and reduced LV function. For oxidative stress, several of these relationships are also supported by essentiality evidence, including links to cardiomyocyte cell death, inflammation, mitochondrial dysfunction, pro-hypertrophic signaling, cardiac fibrosis, and cardiac hypertrophy. This is further supported by drug-induced cardiotoxicity evidence, where mitochondrial injury and ROS-related pathways are well-recognized contributors to cardiomyocyte dysfunction and heart failure outcomes.^21–23^ Thus, the network supports oxidative stress and mitochondrial dysfunction as central propagating events within environmental chemical-induced cardiotoxicity pathways while recognizing that their apparent centrality is also shaped by the structure of the available literature.

The KER-level temporal layer further contextualizes how these stress-related events are positioned within the network. Oxidative stress and mitochondrial dysfunction were observed within acute exposure windows, whereas inflammatory amplification, prohypertrophic signaling, and remodeling-related KERs were more prominent in subacute and chronic categories. Although this does not establish quantitative biological timing, it provides time-scale context for arranging the final AOP sequence, supporting a pattern in which early stress and mitochondrial perturbations are followed by injury and inflammatory KEs, with longer-duration evidence supporting remodeling and functional decline. The close temporal overlap between oxidative stress and mitochondrial dysfunction is also consistent with bidirectional feed-forward interactions shown in the essentiality evidence network. Overall, the final network provides an evidence-weighted sequence-of-events framework for environmental cardiotoxicity, in which general stress-response KEs acquire cardiotoxic relevance through their repeated and partly essentiality-supported connections to cardiac injury, remodeling, and ventricular dysfunction.^14,24,25^

While the network captures many intermediate drivers of cardiotoxicity with high evidence, few MIEs were robustly supported. The most frequently observed MIE was inhibition of mitochondrial electron transport chain complexes (I–V; strongest for complex IV), followed by AhR activation and direct redox-related ROS generation. Other proposed MIEs (e.g., receptor-mediated signaling, metabolic disruption, electrophilic covalent binding) were rarely reported or absent, likely reflecting a literature focus on downstream phenotypes. This evidence distribution limits completeness of the upstream (MIE) layer and yields comparatively denser coverage of intermediate and downstream processes in the current network. Given growing recommendations to recognize cardiotoxicity as a standalone regulatory end point,^7^ future studies should more systematically identify and report diverse cardiotoxicity-relevant MIEs to strengthen AOP coverage.

The prominence of structural cardiac effects/cardiac remodeling (including hypertrophy and fibrosis) and LV dysfunction as AOs in our data-driven AOP network closely aligns with evidence from recent epidemiological studies. A systematic review and meta-analysis of human observational studies demonstrated that exposure to air pollutants (e.g., PM_2_._5_, PM_10_, NO_2_) and heavy metals is associated with early abnormalities in the myocardial structure and function, including both systolic and diastolic LV dysfunction.^12^ Notably, the meta-analysis of epidemiological data highlights these end points as early markers of heart failure risk and recommends their inclusion in future longitudinal and case–control studies. This concordance strengthens the biological plausibility and translational value of our network and illustrates how mechanistic AOP frameworks can complement population-based evidence to confirm causal relationships between environmental chemical exposures and cardiac disease progression.

In contrast to structural cardiac effects, electrophysiological AOs, such as cardiac arrhythmia or disruption of calcium homeostasis, were comparatively underrepresented in the final network despite being commonly investigated. This underrepresentation likely reflects a combination of factors, including end point coverage in the environmental cardiotoxicity literature and the exclusion of observations occurring under strongly adverse exposure conditions during quality filtering. Consequently, applying robustness thresholds (and, separately, excluding observations under strongly adverse exposure conditions) can preferentially reduce electrophysiology-related connectivity, even when some electrophysiology-related nodes remain represented in the network. Threshold sensitivity analysis specifically tracking electrophysiology-related KEs and their associated edges across increasing minimum-support cutoffs (Supporting Information 1, Figure S9) showed that electrophysiology retention declined progressively with stricter thresholds, consistent with the interpretation that electrophysiology-related relationships are supported by fewer repeated observations and are thus more sensitive to robustness filtering than the stress-remodeling core. This observation is notable because electrophysiological liabilities are a primary concern in drug-safety evaluation, where hERG blockade, QT-interval prolongation, and torsade-depointes risk dominate cardiotoxicity screens.^26^ The weak signal in our environmentally focused data set therefore suggests different hazard priorities: while electrophysiological disturbances may be prominent in drug-induced cardiotoxicity, they may be less relevant for environmental chemical exposures at realistic or regulatory-relevant concentrations. This finding also underscores the importance of applying cytotoxicity thresholds (in vitro) and well-being criteria (in vivo) when interpreting electrophysiological outcomes and highlights the need for end point-specific test strategies that reflect the distinct risk profiles of pharmaceuticals versus environmental pollutants.

### Environmental Stressors Converge on Shared Pathways

To explore whether recurrent mechanistic patterns point to prototypical environmental stressors, we next examined the chemical-KE connectivity. Chemicals with extensive study coverage, such as TCDD, cadmium, arsenic, PM_2_._5_, and bisphenol A, showed links to multiple high-frequency KEs, notably oxidative stress, inflammation, and mitochondrial dysfunction. These associations are consistent with those of the broader literature. Cadmium and arsenic exposures have been tied to oxidative stress-mediated cardiovascular morbidity in epidemiological and experimental work,^27,28^ while PM_2_._5_ has been shown to drive cardiac remodeling through inflammatory and redox mechanisms.^29,30^ Notably, the same chemicals identified as key contributors to myocardial abnormalities in our previous epidemiological review, PM_2_._5_, NO_2_, lead, cadmium, and arsenic, also emerged as mechanistically well-supported stressors in our in vitro and in vivo evidence mapping. This convergence of epidemiological and mechanistic evidence not only strengthens the case for regulatory attention to these pollutants but also underscores the validity of our integrative approach for identifying priority stressors and relevant mechanistic pathways in environmental cardiotoxicity.

At the same time, the absence of a strong, stable chemical clustering signal should not be interpreted as evidence that stressors are mechanistically indistinguishable. In a literature-derived data set like this, two structural features naturally compress chemical separability: (i) uneven study coverage across chemicals and (ii) high-prevalence end points that are used repeatedly across many stressors and therefore dominate patterns. Broad convergence on shared processes is itself a substantive feature of the environmental cardiotoxicity evidence base and aligns with the stressor-agnostic backbone captured by the AOP network. To avoid overinterpreting patterns driven primarily by study frequency and end point popularity, we additionally examined row-normalized chemical-KE and group-KE profiles with FDR-adjusted enrichment. The enrichment patterns highlight stressor-linked tendencies that align with prior mechanistic expectations (e.g., AhR-linked responses for TCDD) and point to a limited set of chemical- or class-associated end points that appear sufficiently replicated to rise above the background of shared stress responses.

In contrast, many pesticides, industrial chemicals, and engineered nanoparticles were connected to only a handful of KEs and were represented by very few studies, precluding confident assessment of their cardiotoxic potential. This data sparsity may reflect research gaps and publication bias rather than lack of hazard, underscoring the need for targeted mechanistic studies on undercharacterized but commonly occurring chemicals. These findings further emphasize the utility of a chemical-agnostic, evidence-based mapping approach: it can highlight well-supported chemical– mechanism relationships while also pointing to substances in need of further investigation to clarify their cardiotoxicity profiles.

Modulating factors such as high-fat diet and pre-existing cardiovascular conditions further indicate that susceptibility and KE intensity are context-dependent, but uneven reporting limits quantitative integration of modifiers into the network at present.

### The Consensus Network Links Early Stress Responses to Time-Dependent Cardiac Remodeling and Dysfunction

Building on the evidence-weighted candidate KER subnetworks, we derived a final consensus AOP network representing the most consistently supported cardiotoxicity-related event sequence in the curated evidence base (Figure 6). This consolidation integrated four complementary considerations: empirical support, essentiality-supported directionality, biological plausibility, and KER-specific time scale context. The final network is therefore not a direct frequency plot of all extracted relationships but a condensed synthesis of recurrent, biologically interpretable KERs arranged according to their evidence support and temporal context. This consensus network highlights oxidative stress and mitochondrial dysfunction as stress-response and propagation KEs, with inflammation acting as a recurrent propagating and amplifying process. These events connect upstream molecular and subcellular perturbations, including mitochondrial electron transport chain inhibition, direct redox-mediated ROS production, and AhR activation, to downstream cardiac injury and functional outcomes. In the final sequence, early stress and mitochondrial perturbations are followed by cardiomyocyte injury/death and inflammatory signaling, with longer-duration evidence supporting prohypertrophic and pro-fibrotic signaling, cardiac remodeling, and reduced LV function.

**Figure 6:**
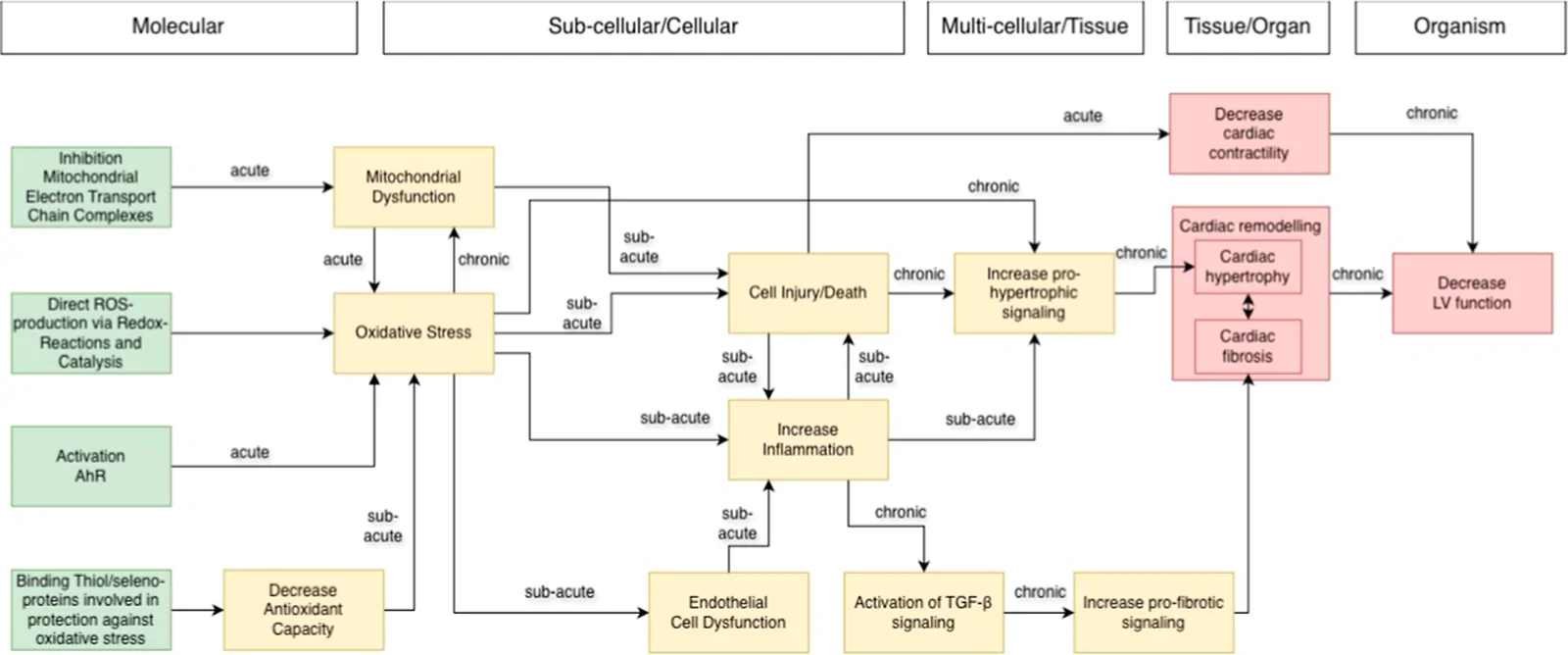
Final time-resolved AOP network for the most frequently observed cardiotoxicity mechanisms induced by environmental chemicals. KER-level annotation captures temporal context more effectively than KE-level timing.

A key methodological finding is that temporal interpretation was more informative at the KER level than at the KE level. While we initially hypothesized that exposure durations might follow a general trend from acute MIEs to chronic AOs, this pattern did not hold when KEs were examined in isolation. In fact, several KEs, regardless of their biological level, were observed across a wide range of exposure durations, making temporal interpretation at the KE level inconsistent and potentially misleading as it ignores the context-specific, relational nature of biological timing. However, when exposure duration was analyzed per KER, distinct and interpretable patterns emerged. Each KER, even when it involves the same KE, is associated with a characteristic duration that reflects the temporal spacing of the upstream and downstream events. This relationship-level resolution revealed a more accurate picture of cardiotoxic progression, highlighting, for instance, that certain AOs, such as decreased myocardial contractility, can occur after acute exposures, while some upstream or intermediate events (e.g., chronic inflammation or pro-fibrotic signaling) may require subacute or chronic exposure to manifest. Thus, the temporal layer provided a data-derived constraint for arranging the final AOP sequence.

This relationship-level temporal structure extends conventional qualitative AOP representation by embedding literature-derived time-scale information directly at the KER level. Although the AOP Developers’ Handbook recognizes time scale and temporal concordance as relevant to KER evaluation,^31^ and the AOP-Wiki allows temporality to be discussed in supporting evidence, qualitative AOP resources do not yet provide a standardized way to represent when transitions are typically observed.^32^ KER-level annotation addresses this gap while preserving the modularity of KEs, which may participate in different relationships across different time scales. It also has practical value for follow-up study design and Integrated Approaches for Testing and Assessment (IATA), as measurement end points and exposure or observation windows can be aligned with the specific KE transition under evaluation.

At the same time, the temporal categorization remains a pragmatic approximation based on study design windows and does not account for chemical- or species-specific toxicokinetics and internal dose. As more longitudinal and time-course data sets become available, this literature-derived time-scale layer could be refined toward more quantitative time concordance. In its current form, the network provides an evidence-weighted, time-resolved framework for interpreting environmental chemical-induced cardiotoxicity and for identifying KERs where additional targeted experimental evidence would most improve confidence.

## Supplementary Material







## Notes

Supplement 1, supplementary methods, references, figures, and tables; species- and system-specific exposure-duration thresholds; network sensitivity analyses; and species/system and chemical-KE enrichment analyses (PDF)
Supplement 2, curated AOP data sets including KEs, KERs, essentiality relationships, study metadata, chemical associations, and exposure-duration annotations (XLSX)
Supplement 3, interactive HTML KE-network visualizations (ZIP)
