# Supplementary material for: A Data-Driven Approach for the Development of a Time-Informed Adverse Outcome Pathway Network for the Cardiotoxicity of Environmental Chemicals
Source: Environ Sci Technol. 2026 Oct 2;60(41):29273–84. doi: 10.1021/acs.est.6c00396 (PMC13689818; doi:10.1021/acs.est.6c00396)
Supplement: Supplementary file 1 [file es6c00396_si_001.pdf]

Supplementary 1

A Data-Driven Approach for the Development of a Time-informed Adverse Outcome Pathway Network for Cardiotoxicity of Environmental Chemicals

Alexandra Schaffert[1,2,3]#, Sivakumar Murugadoss[4]#, Tom Roos[5]#, Nunzia Linzalone[6], Gabriele Donzelli[6], Ronette Gehring[5], Birgit Mertens[7]†*, Martin Paparella[2]†*

[1] Finnish Hub for Development and Validation of Integrated Approaches (FHAIVE), Faculty of Medicine and Health Technology, Tampere University, 33100 Tampere, Finland
[2] Institute of Medical Biochemistry, Medical University Innsbruck, 6020 Innsbruck, Austria
[3] Tampere Institute for Advanced Study, Tampere University, 33100 Tampere, Finland
[4] Health Effects Laboratory, Department of Environmental Chemistry and Health Effects, NILU The Climate and Environmental Research Institute, 2007 Kjeller, Norway
[5] Department of Population Health Sciences, Institute for Risk Assessment Sciences (IRAS), Faculty of Veterinary Medicine, Utrecht University, 3584 Utrecht, the Netherlands
[6] Institute of Clinical Physiology, National Research Council, 56124 Pisa, Italy
[7] Scientific Department of Chemical and Physical Health Risks, Sciensano, 1050 Ixelles, Belgium

# These authors contributed equally to this work and share first authorship
† These authors contributed equally to this work and share last authorship
** Correspondance: M. Paparella martin.paparella@i-med.ac.at, B. Mertens Birgit.Mertens@sciensano.be*

This Supplementary includes 13 pages, 9 figures, and 3 tables.

Table S1: Overview of most relevant criteria extracted during the systematic review. 3
Table S2: Species- and system-specific reference exposure durations and classification thresholds used for exposure time categorization. 5
Table S3: Network metrics and hub identity across observation thresholds. 9

Figure S1: Biological models used in studies included for AOP extraction. 6
Figure S2:Species and experimental-system distribution of evidence across the top 40 KERs. 6
Figure S3:Threshold sensitivity analysis of the AOP network structure. 7
Figure S4:Retained and removed KEs in the AOP network based on minimum observation thresholds ranging from ≥1 to ≥6 references per KER. 8
Figure S5: Relative frequency of KE organisation levels per duration. 9
Figure S6: Duration classes for in vivo (A) and in vitro (B) KEs on different biological organisation levels. 9
Figure S7: Chemical-KE entry-normalized profiles. Heatmap shows, for each chemical (row), the fraction of extracted chemical-KE entries assigned to each KE (columns), i.e. an entry-normalized KE profile. 10
Figure S8: Chemical group-KE entry-normalized profiles. 11
Figure S9: Threshold sensitivity analysis with electrophysiology tracking...12

Methodology

AOP data extraction and KE vocabulary harmonisation

Data from both extractions were made sure to align for consistency. For each study, key outcomes were extracted, alongside the methods used to assess these outcomes (e.g., qPCR), a description of outcomes (e.g., gene expression of pro-inflammatory cytokines was increased), and the corresponding KE (e.g., Inflammation). If multiple assays within the same experiment assessed the same KE, multiple entries were recorded but counted as a single KE occurrence for support analyses. In studies including both *in vitro* and *in vivo* experiments, KEs were extracted separately for each experimental system. Only outcomes with statistically significant changes were considered as extractable KEs and no-effect outcomes excluded. This is noted as a limitation, as their absence precludes evaluation of potential opposing effects, although such reporting is generally limited in research and therefore represents a bias in itself. For each KE, it was recorded whether the outcome occurred only under (cyto)toxic doses or not (Table S1).

In cases where essentiality of a KE was tested, such as, e.g., through the use of inhibitory agents targeting an upstream KE, the name of the upstream KE and the testing agent were recorded alongside the downstream KE. If a modulating factor (e.g., sex, diet, pre-existing conditions) significantly affected the outcome, this information was also documented in relation to the corresponding KE.

KE terminology was harmonised in two rounds. In the first extraction round, mechanistic outcomes were mapped to an initial controlled vocabulary of predefined KE terms derived from cardiotoxicity-relevant AOP-Wiki entries and epidemiologically informed AO definitions. Where none of the predefined KEs adequately captured the reported effect, extractors assigned new candidate KE terms directly from the study-derived observation. Importantly, this procedure resulted in an empirically expanded KE library in which the majority of KEs captured in the evidence base were not covered by the predefined AOP-Wiki-derived terms. The predefined KE vocabulary was intentionally restricted to three cardiotoxicity-relevant AOP-Wiki entries present at the time of the extraction: AOP 433 [1], AOP 261 [2], and 262 [3]; broader cardiovascular AOPs were not included because they fell outside the defined study scope. Following consolidation and harmonisation of terminology across all extracted observations, a second extraction round re-mapped all KE annotations using the standardised KE set to ensure consistency and reduce synonym-driven fragmentation.

AOP data extraction was performed equally by three authors (A.S., S.M., and T.R.). Extracted entries and KE assignments were cross-checked by A.S. and S.M., and uncertain cases were resolved by discussion among the extracting authors.

Table S1: Overview of most relevant criteria extracted during the systematic review.

**Extraction item**	**Explanation**
Biological system	Animal type, strain, life stage, or cell type
Chemical information	Chemical name, type, CAS number, purity, source, mixture components
Exposure scenario	Exposure duration, route, dose, number of concentrations tested
KE name	Best fitting name for potential cardiotoxicity-related KE (harmonized among data)

Essentiality assessment	Essentiality of a KE and how it was tested, e.g., addition of antioxidant which inhibits KE “Increase oxidative stress” lead to diminishing of downstream KE “Increase cell death/cell injury”. Thus, during extraction of KE “Increase cell death/cell injury”, essentiality testing was entered in the row and “Increase oxidative stress” was entered as upstream connected essential KE.
(Cyto)toxicity	*In vitro*: Yes/No for significant cytotoxicity at KE-relevant dose, or if cytotoxicity was not tested<br>*In vivo*: Yes/No for reported cases of mortality or significant decrease in body weight compared to the control, or if body weight was not reported at the end of experiment
Modulating factors	Factors which affected the intensity of the KE (e.g., sex, diet, preexisting conditions)
Method/assay	Experimental method or assay which was used to assess the KE
Outcome	Description of the outcome associated with the KE
Risk of Bias assessment	Risk of Bias was assessed for each study, consisting of 11 questions, including 2 key questions.

Essentiality-supported directionality

Directionality was assigned only where essentiality evidence was available. Essentiality-supported KERs were defined as relationships for which targeted perturbation of an upstream KE resulted in a measurable change in a downstream KE. Examples included pharmacological inhibition, antioxidant intervention, receptor modulation, genetic manipulation, or other targeted experimental perturbations. The AOP Developers' Handbook describes essentiality as evidence that modification or prevention of an upstream KE affects downstream KEs or the AO. KERs without essentiality evidence were retained as empirically supported relationships if they met the support and plausibility criteria but were not interpreted as directionally or causally demonstrated.

KER time annotation

For in vivo mammalian and avian studies, Acute exposure was defined as a single administration with a total duration below the species-specific acute threshold (typically up to 1 day). Dosing regimen was inferred from the study description. Repeated or continuous dosing, even if shorter than the acute threshold, was therefore classified as Subacute. Subacute, Subchronic, and Chronic categories covered increasingly longer exposure windows up to and beyond the species-specific cut-offs corresponding to subacute (for example 28 days in rodents), subchronic (for example 90 days in rodents), and chronic exposures (multi-month or lifetime), respectively. For aquatic and invertebrate in vivo models (zebrafish, tadpoles, Drosophila), acute windows were adjusted to reflect shorter life cycles and typical guideline practice. Continuous exposures of a few days were still classified as Acute when they remained below the species-specific acute threshold; longer exposures were assigned to Subacute, Subchronic, or Chronic according to the cut-offs listed in Supplementary Table S1. For in vitro studies, exposure duration classification was based solely on incubation time, independent of dosing frequency. Exposures were categorised as Acute (up to 3 days), Subacute (more than 3–14 days), Subchronic (more than 14–28 days), or Chronic (more than 28 days), reflecting common practice in mechanistic and omics-based assays. The full category classification and all species-specific thresholds were documented in Supplement 1, Table S2.

Table S2: Species- and system-specific reference exposure durations and classification thresholds used for exposure time categorization.

Experiment Type	Species category	Acute ≤	Subacute >…≤	Subchronic >…≤	Chronic >	Acute requires single dose?	Short continuous allowed as Acute?	Justification
in_vivo	Mice	1 d	28 d	90 d	90 d	Yes	No	OECD acute oral TGs are single-dose with 14-d observation; 28- and 90-day repeated-dose define subacute/ subchronic (TGs 420, 423, 407, 408)
in_vivo	Rats	1 d	28 d	90 d	90 d	Yes	No	Same rationale: acute = single dose; TG 407 (28 d) and TG 408 (90 d) for longer windows
in_vivo	Zebrafish (incl. embryos)	4 d	21 d	60 d	60 d	No	Yes	Aquatic “acute” tests are 24–96 h (fish acute TG 203; FET TG 236); ELS TG 210 extends to early juvenile → places 21–60 d bounds
in_vivo	Other: Drosophila melanogaster	4 d	21 d	60 d	60 d	No	Yes	No OECD TG; ~10 d generation time and ~60–70 d lab lifespan justify ≤4 d “acute” and >21–60 d subchronic bounds [4]
in_vivo	Swine (incl. pigs)	1 d	28 d	91 d	91 d	Yes	No	Non-rodent general tox practice per ICH M3(R2): ~13 wk subchronic; ≥6–9 mo chronic. We set the boundary at 13 wk
in_vivo	Chicken (incl. broilers & eggs)	1 d	70 d	140 d	140 d	Yes	No	Acute oral TG 223 = single dose with 14-d observation; avian reproduction TG 206 collects eggs for ~10 wk = 70 d, anchoring higher categories
in_vivo	Rabbits	1 d	28 d	91 d	91 d	Yes	No	Treat as non-rodent per ICH M3(R2) (subchronic ≈13 wk; chronic ≥6–9 mo)
in_vivo	Other: Tadpoles	4 d	21 d	60 d	60 d	No	Yes	Amphibian Metamorphosis Assay TG 231 is a 21-day exposure; ≤4 d follows aquatic “acute” convention; >21–60 d subchronic; >60 d chronic
in_vivo	Quails (incl. eggs)	1 d	70 d	140 d	140 d	Yes	No	Same avian anchors as chicken: TG 223 (single dose + 14 d obs) and TG 206 (~10 wk egg-collection)
in_vivo	Other: Marmosets	1 d	28 d	91 d	91 d	Yes	No	Non-rodent primate durations per ICH M3(R2); many chronic studies are 26–52 wk
in_vitro	all species	3 d	14 d	28 d	28 d	No	Yes	Validated assays use 24–72 h exposures (e.g., KeratinoSens™ 48 h TG 442D; h-CLAT ~24 h TG 442E). GIVIMP frames exposure as method-defined

Expert refinement of consensus AOP network

Biological plausibility review considered whether each upstream-downstream KE pair was consistent with established knowledge of cardiac physiology and pathophysiology and with AOP construction logic. This included whether a generally accepted structural or functional relationship existed between the two KEs, whether the direction of the relationship was compatible with the assigned biological levels, and whether intermediate cellular or tissue-level events were not bypassed in a way that made the relationship mechanistically non-interpretable, in this case plausible intermediate events were identified from the candidate networks. KER-specific temporality was considered during consensus network derivation as

contextual support for sequence plausibility. Specifically, time-scale information helped arrange KERs so that relationships predominantly observed after acute exposures were not placed downstream of relationships mainly observed after longer exposure durations. This temporal layer was used to support biological plausibility of the event sequence, not to infer quantitative time-to-event dynamics. The consolidation was led by A.S. and S.M., and discussed with the author team and advisory cardiology experts where needed. Uncertain cases were resolved by consensus discussion among the co-authors.

Figures

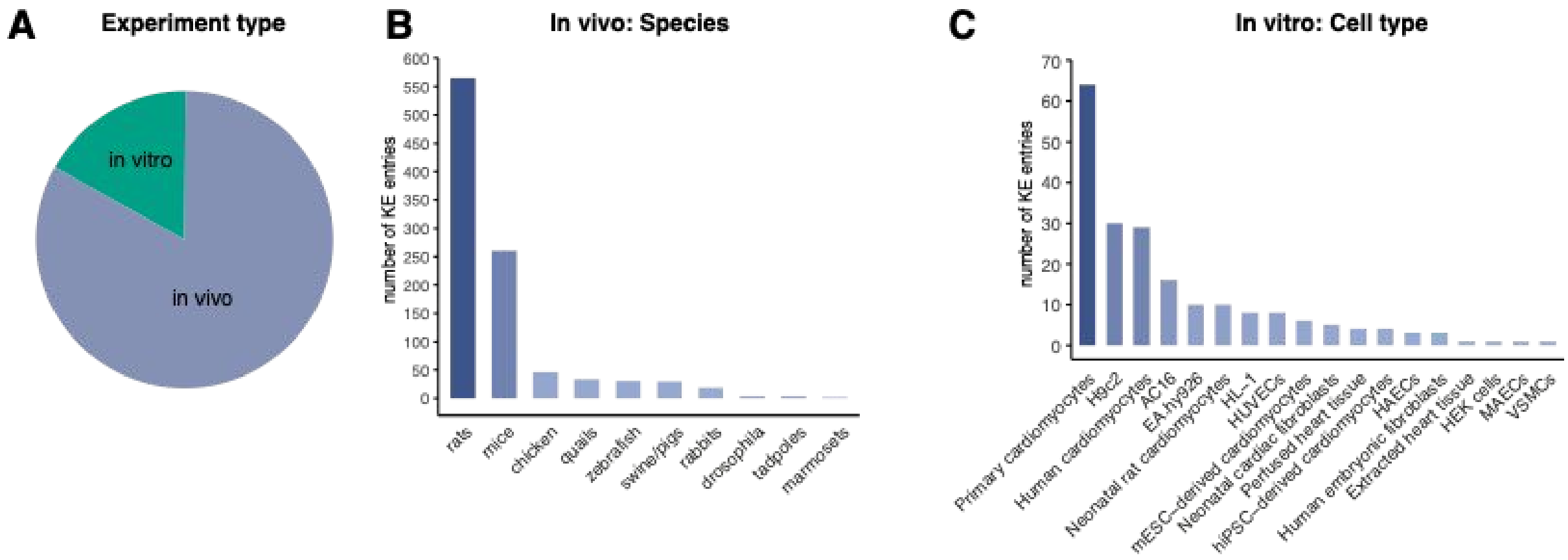

Figure S1: Biological models used in studies included for AOP extraction.
Proportion of KE entries derived from in vivo versus in vitro studies. Species distribution in in vivo KE entries. Cell types used in in vitro KE entries

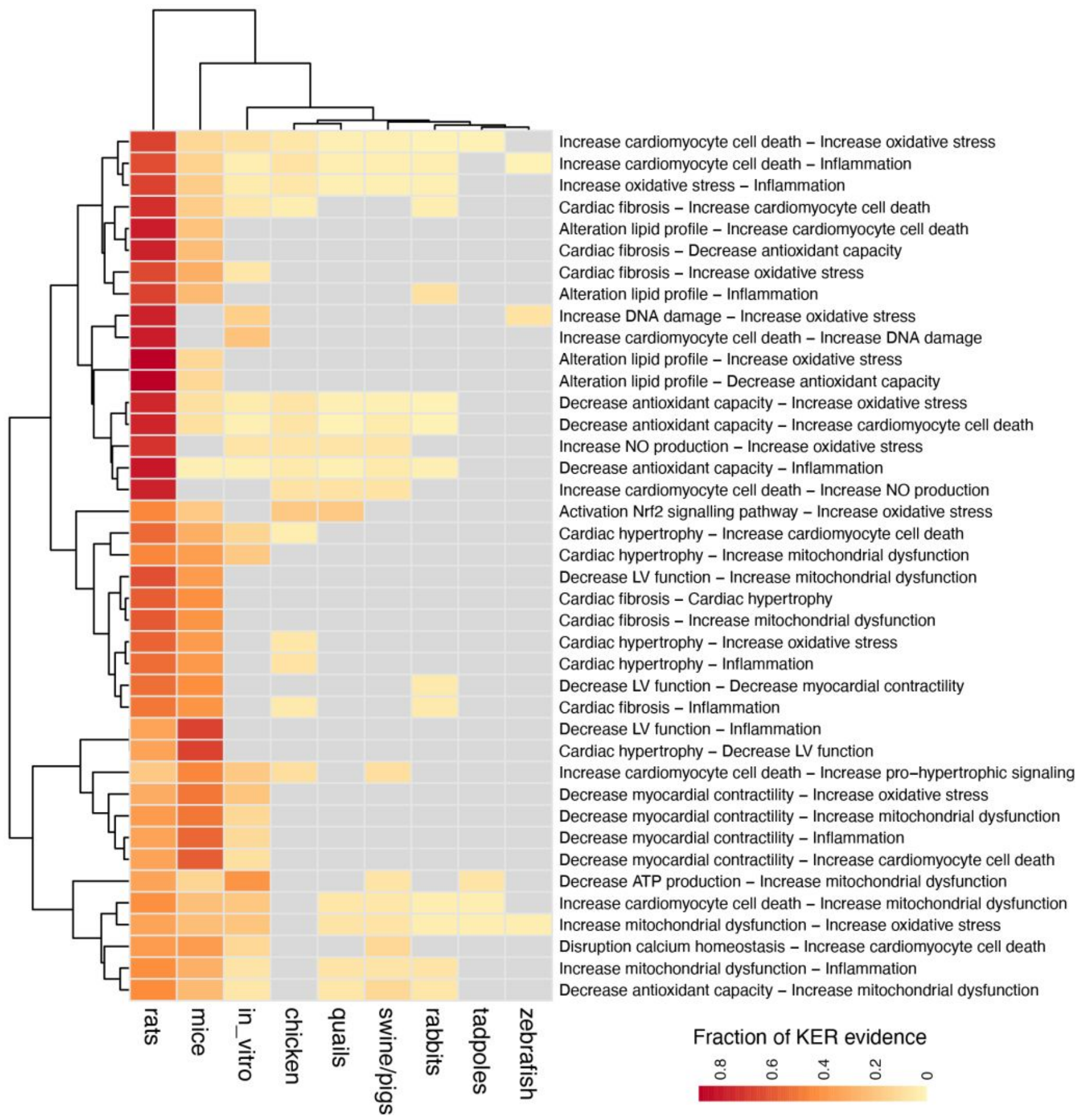

Figure S2:Species and experimental-system distribution of evidence across the top 40 KERs.

Rows show the 40 most frequently observed KERs; columns show in vivo taxa and an aggregated in_vitro evidence category. Cell color indicates the fraction of total evidence per KER contributed by each taxon/system (row-normalized); grey = no detected evidence. Rows/columns are hierarchically clustered.

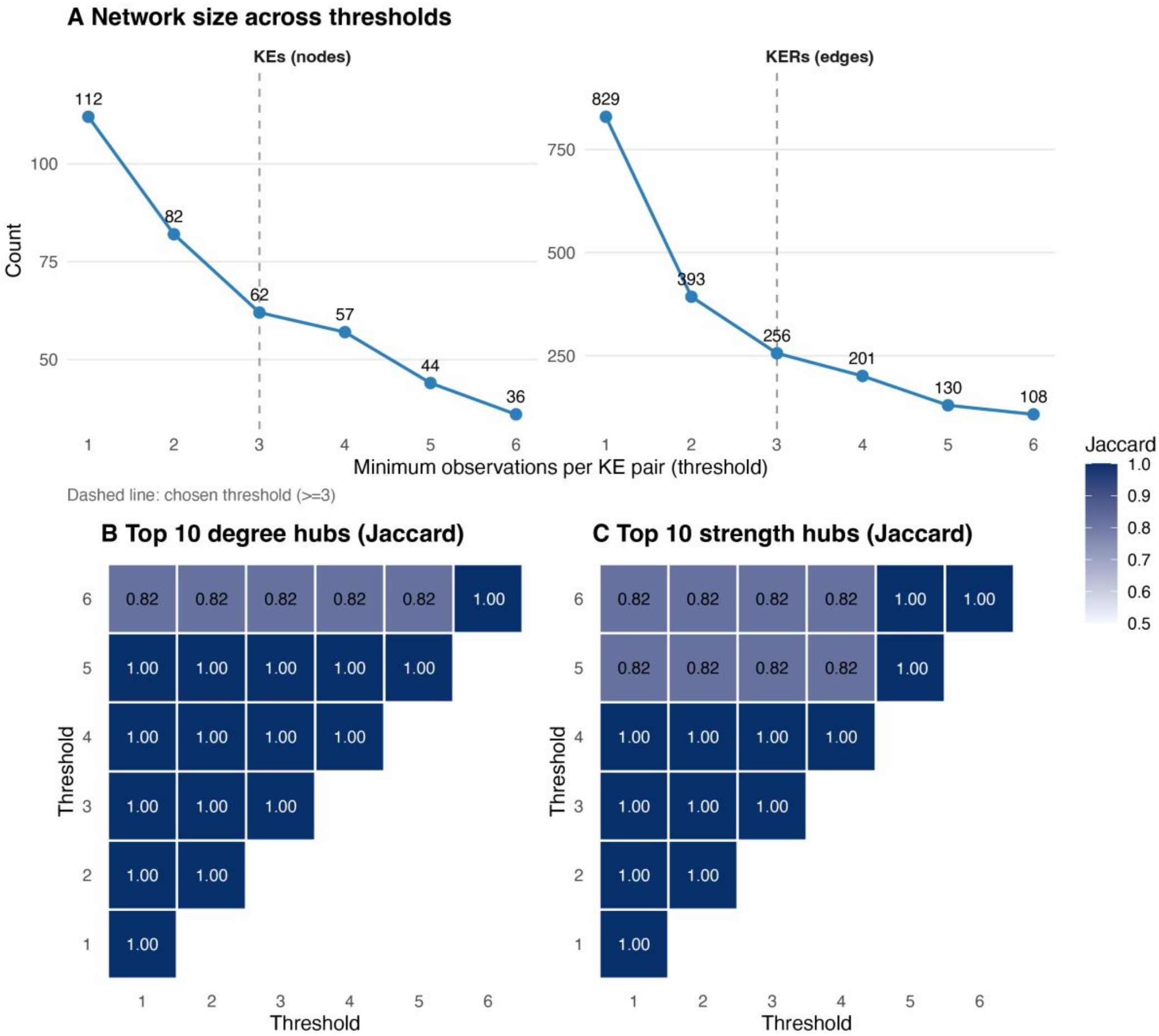

Figure S3:Threshold sensitivity analysis of the AOP network structure.
Networks were constructed at minimum observation thresholds ranging from ≥1 to ≥6 references per KER. (A) Network size as a function of the observation threshold. The number of Key Events (KEs, nodes; left) and Key Event Relationships (KERs, edges; right) decreased monotonically with stricter thresholds; exact counts are shown above each point. The dashed vertical line indicates the threshold (≥3) used in the main analysis. (B, C) Pairwise Jaccard similarity of the top10 hubs across thresholds, computed for unweighted degree (B) and weighted degree/strength (C). Cells show the proportion of shared hubs between each pair of thresholds (Jaccard = |A ∩ B| / |A ∪B|); diagonal entries are 1.0 by definition. Top10 degree hubs were identical between thresholds ≥1 and ≥4 (Jaccard = 1.0) and showed a single boundary substitution at ≥5 and ≥6 (Jaccard = 0.82). Top10 strength hubs were identical across all thresholds from ≥2 to ≥6 (Jaccard = 1.0). The high stability across thresholds indicates that the central mechanistic structure of the network is robust to reasonable changes in the chosen observation threshold.

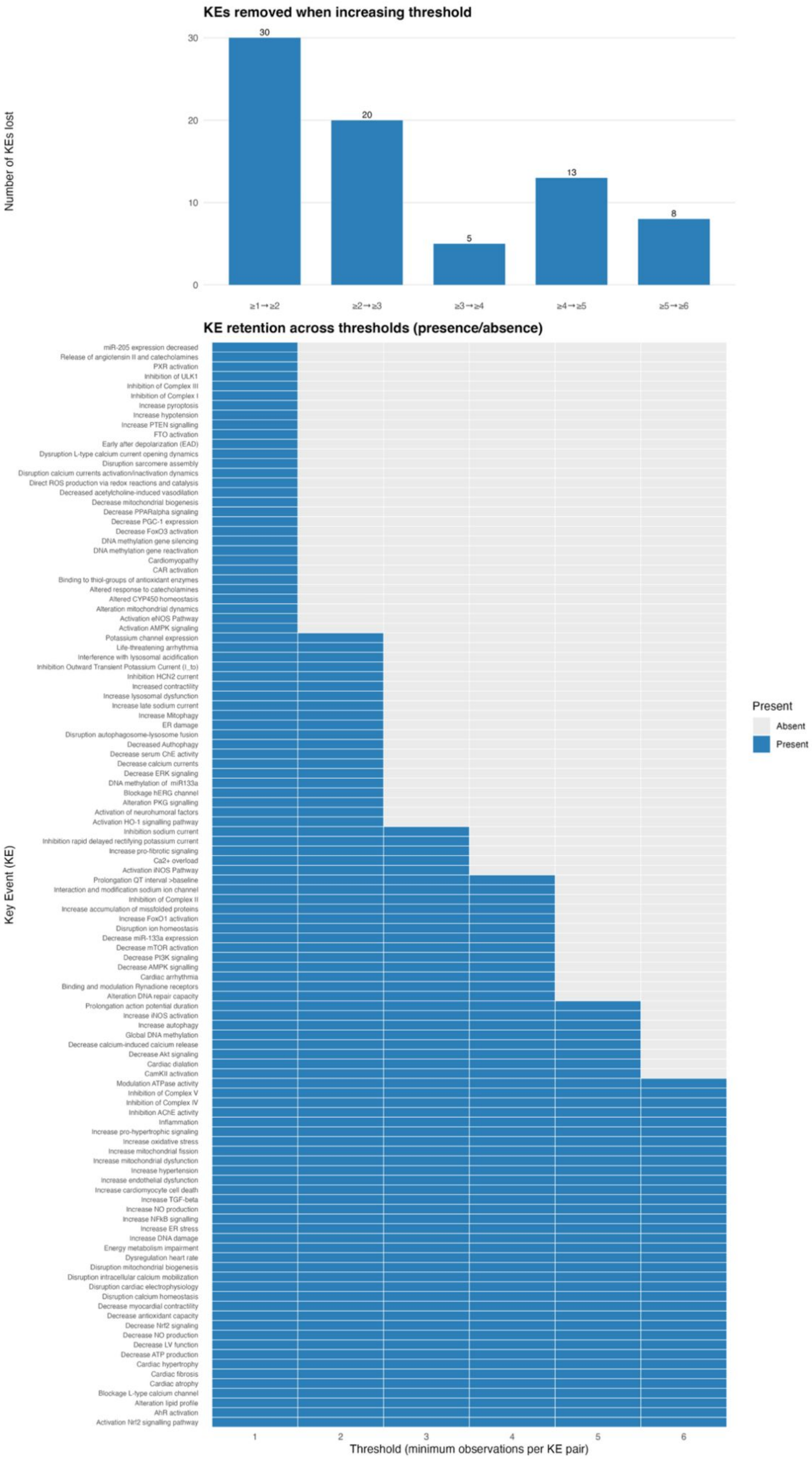

Figure S4:Retained and removed KEs in the AOP network based on minimum observation thresholds ranging from ≥1 to ≥6 references per KER.

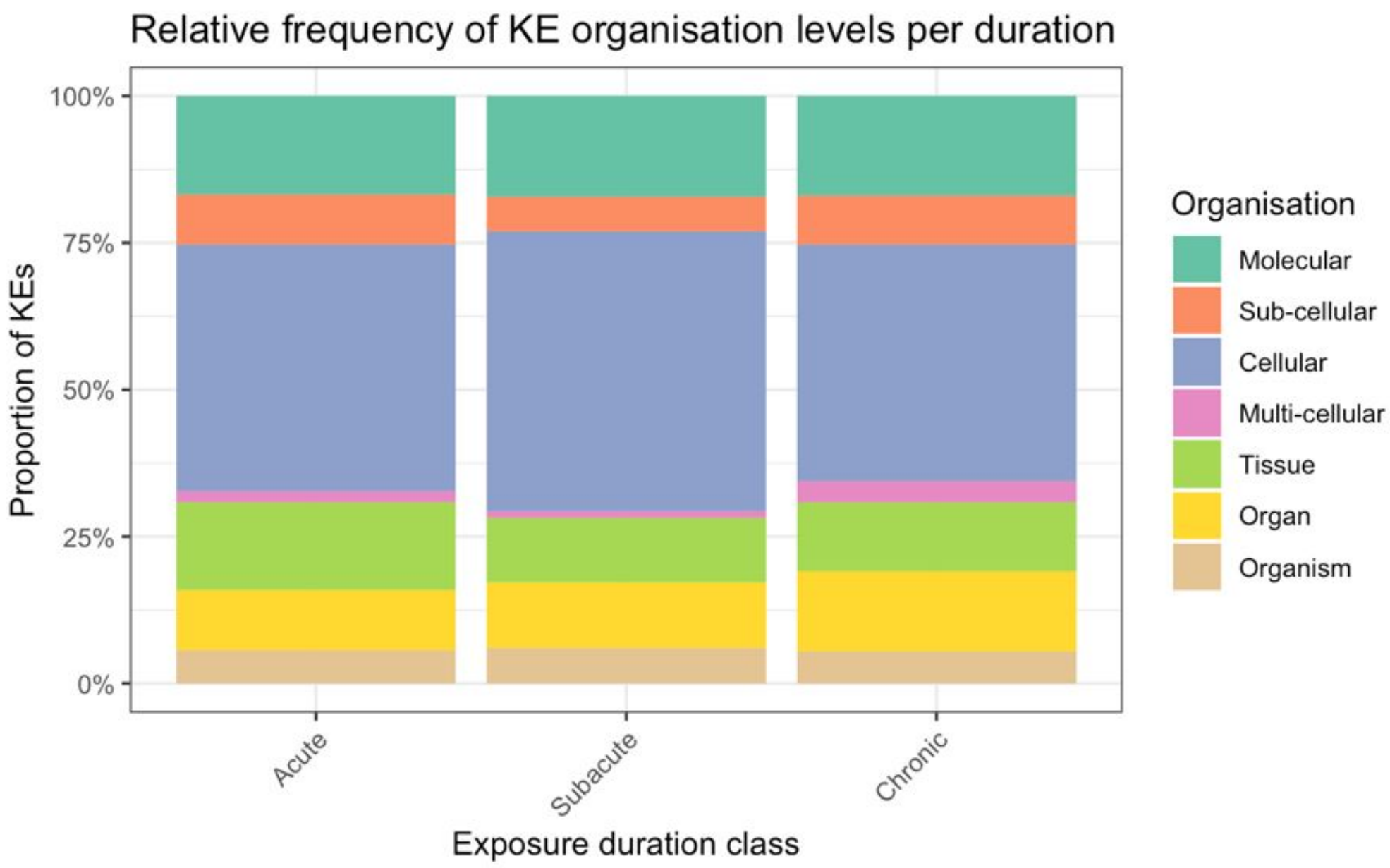

Figure S5: Relative frequency of KE organisation levels per duration.

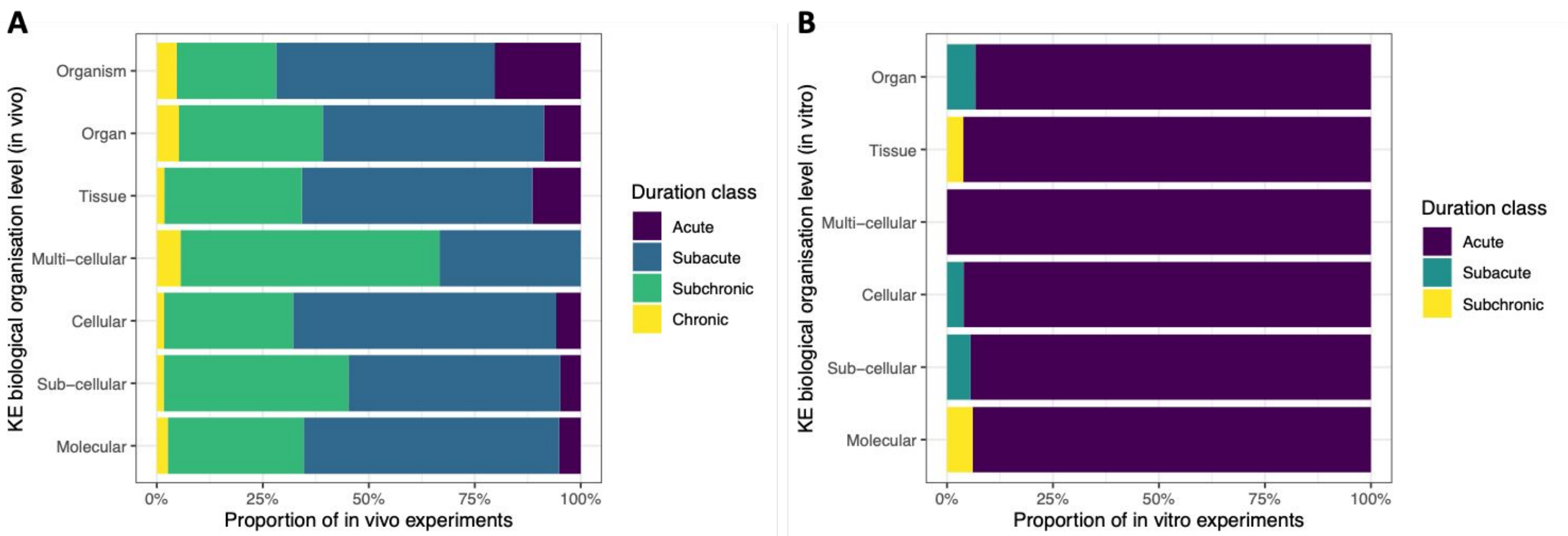

Figure S6: Duration classes for in vivo (A) and in vitro (B) KEs on different biological organisation levels.

Table S3: Network metrics and hub identity across observation thresholds.
For each minimum observation threshold (≥1 to ≥6 per KE pair), the table reports the top-10 hub KEs ranked by unweighted degree and weighted degree (strength).

Threshold	Top-10 degree hubs	Top-10 strength hubs
≥1	Increase cardiomyocyte cell death; Increase oxidative stress; Inflammation; Decrease antioxidant capacity; Increase mitochondrial dysfunction; Cardiac hypertrophy; Decrease myocardial contractility; Cardiac fibrosis; Decrease LV function; Increase pro-hypertrophic signaling	Increase cardiomyocyte cell death; Increase oxidative stress; Decrease antioxidant capacity; Inflammation; Increase mitochondrial dysfunction; Cardiac hypertrophy; Cardiac fibrosis; Decrease myocardial contractility; Decrease LV function; Increase pro-hypertrophic signaling
≥2	Increase cardiomyocyte cell death; Increase oxidative stress; Increase pro-hypertrophic signaling; Decrease antioxidant capacity; Cardiac hypertrophy; Decrease myocardial contractility; Inflammation; Increase mitochondrial dysfunction; Cardiac fibrosis; Decrease LV function	Increase cardiomyocyte cell death; Increase oxidative stress; Decrease antioxidant capacity; Inflammation; Increase mitochondrial dysfunction; Cardiac hypertrophy; Cardiac fibrosis; Decrease myocardial contractility; Decrease LV function; Increase pro-hypertrophic signaling
≥3	Increase cardiomyocyte cell death; Increase oxidative stress; Inflammation; Decrease antioxidant capacity; Cardiac hypertrophy; Increase mitochondrial dysfunction; Increase pro-hypertrophic signaling; Decrease myocardial contractility; Decrease LV function; Cardiac fibrosis	Increase cardiomyocyte cell death; Increase oxidative stress; Decrease antioxidant capacity; Inflammation; Increase mitochondrial dysfunction; Cardiac hypertrophy; Cardiac fibrosis; Decrease myocardial contractility; Decrease LV function; Increase pro-hypertrophic signaling

≥4	Increase cardiomyocyte cell death; Increase oxidative stress; Inflammation; Cardiac hypertrophy; Decrease antioxidant capacity; Increase mitochondrial dysfunction; Decrease myocardial contractility; Increase pro-hypertrophic signaling; Decrease LV function; Cardiac fibrosis	Increase cardiomyocyte cell death; Increase oxidative stress; Decrease antioxidant capacity; Inflammation; Increase mitochondrial dysfunction; Cardiac hypertrophy; Cardiac fibrosis; Decrease myocardial contractility; Decrease LV function; Increase pro-hypertrophic signaling
≥5	Increase cardiomyocyte cell death; Increase oxidative stress; Inflammation; Decrease antioxidant capacity; Decrease myocardial contractility; Increase mitochondrial dysfunction; Cardiac hypertrophy; Cardiac fibrosis; Decrease LV function; Increase pro-hypertrophic signaling	Increase cardiomyocyte cell death; Increase oxidative stress; Decrease antioxidant capacity; Inflammation; Increase mitochondrial dysfunction; Cardiac hypertrophy; Cardiac fibrosis; Decrease myocardial contractility; Decrease LV function; Alteration lipid profile
≥6	Increase oxidative stress; Increase cardiomyocyte cell death; Increase mitochondrial dysfunction; Inflammation; Decrease antioxidant capacity; Cardiac hypertrophy; Cardiac fibrosis; Decrease myocardial contractility; Decrease LV function; Alteration lipid profile	Increase cardiomyocyte cell death; Increase oxidative stress; Decrease antioxidant capacity; Inflammation; Increase mitochondrial dysfunction; Cardiac hypertrophy; Cardiac fibrosis; Decrease myocardial contractility; Decrease LV function; Alteration lipid profile

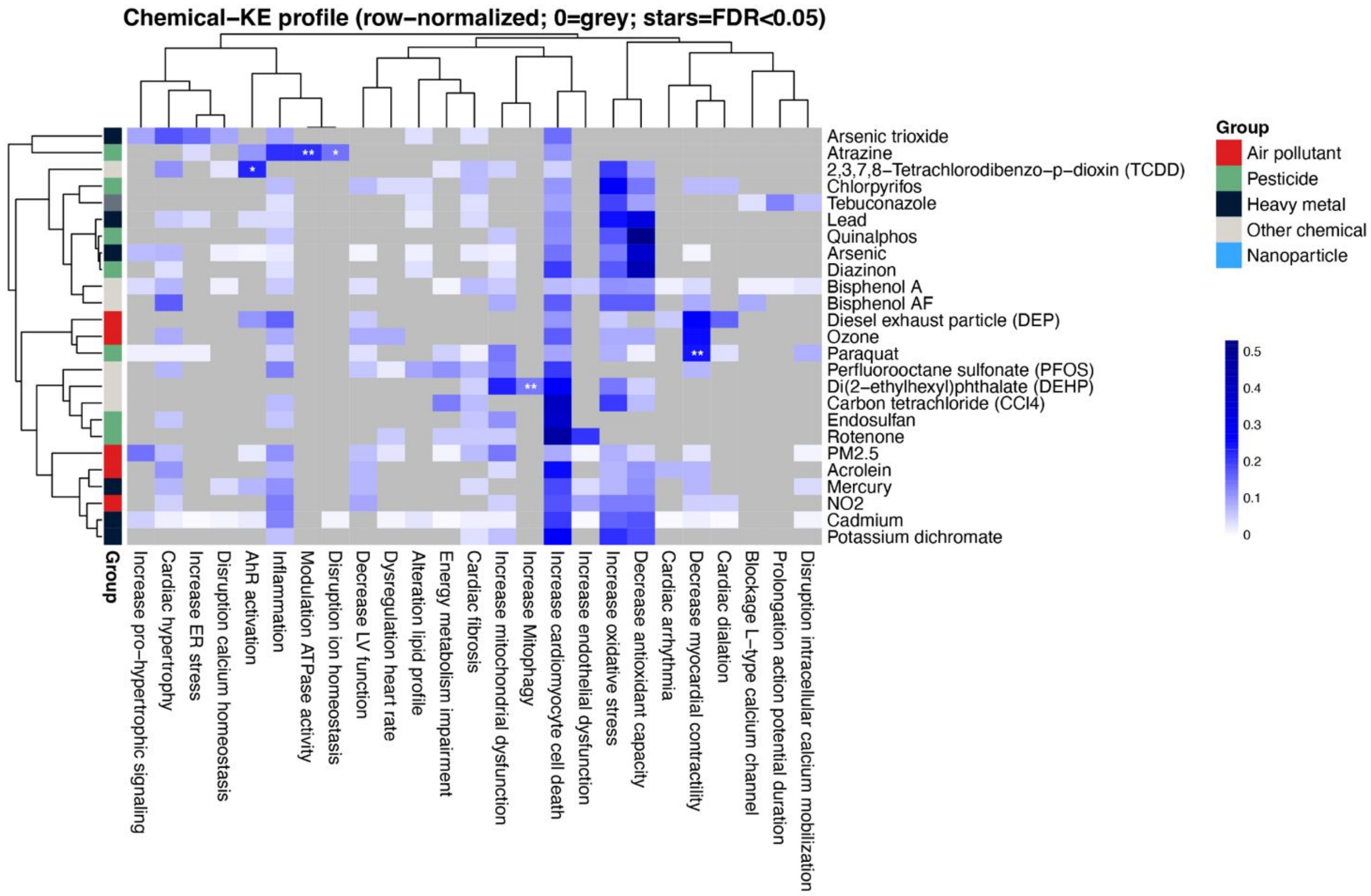

Figure S7: Chemical-KE entry-normalized profiles. Heatmap shows, for each chemical (row), the fraction of extracted chemical-KE entries assigned to each KE (columns), i.e. an entry-normalized KE profile.
Cells with 0 entries are shown in grey to indicate no recorded association in the extracted dataset. Chemical rows and KE columns are hierarchically clustered. The row side bar indicates the assigned chemical class/group. Stars indicate enrichment of a KE for a given chemical (KE present at least once in a study for that chemical) using Fisher's exact test comparing the chemical to all other chemicals, with Benjamini-Hochberg FDR correction within chemical (FDR < 0.05, ** < 0.01, *** < 0.001).*

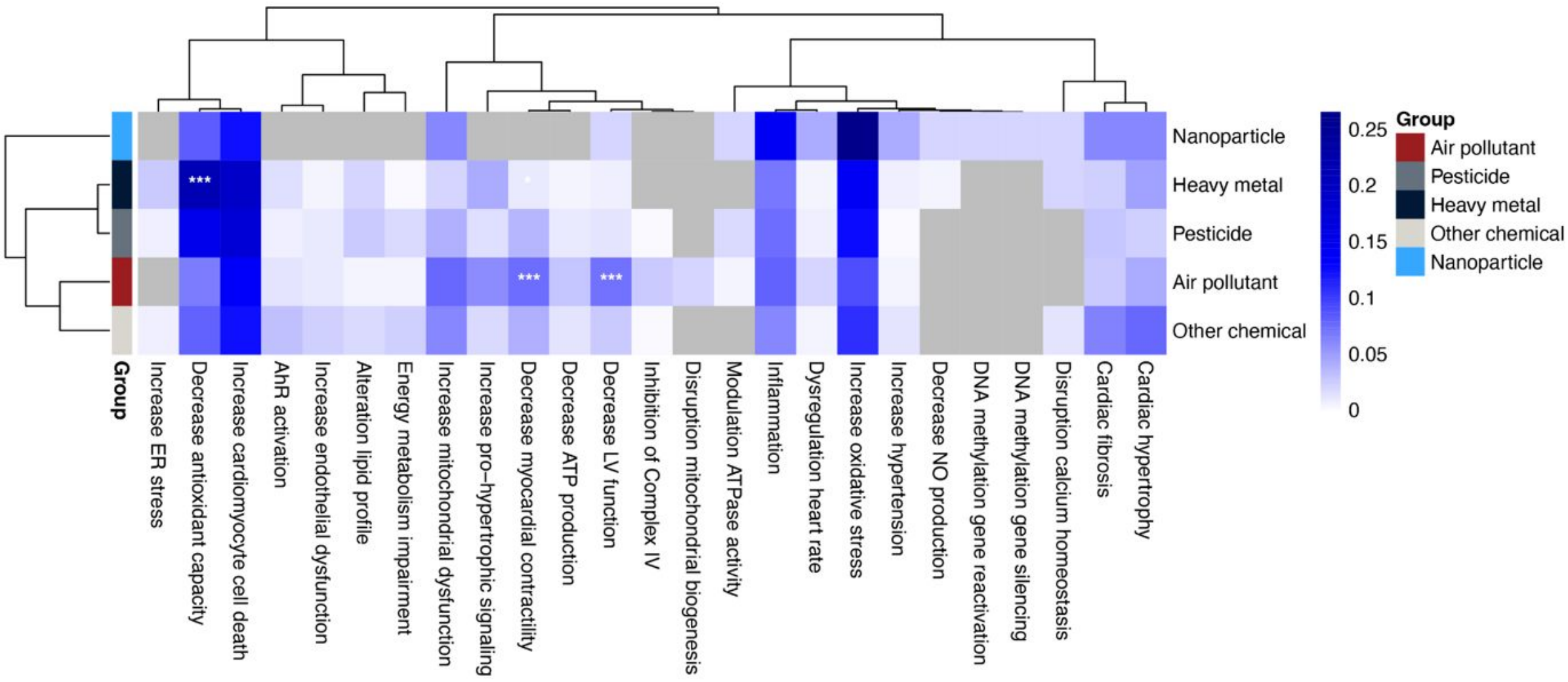

Figure S8: Chemical group-KE entry-normalized profiles.

Heatmap shows, for each chemical group (row), the fraction of extracted group-KE entries assigned to each KE (columns), i.e. an entry normalized KE profile aggregated by group. Cells with 0 entries are shown in grey to indicate no recorded association in the extracted dataset. Group rows and KE columns are hierarchically clustered. Stars indicate study-level enrichment of a KE for a given group using Fisher's exact test (group vs all other groups), with Benjamini-Hochberg FDR correction within group (FDR < 0.05, ** < 0.01, *** < 0.001).*

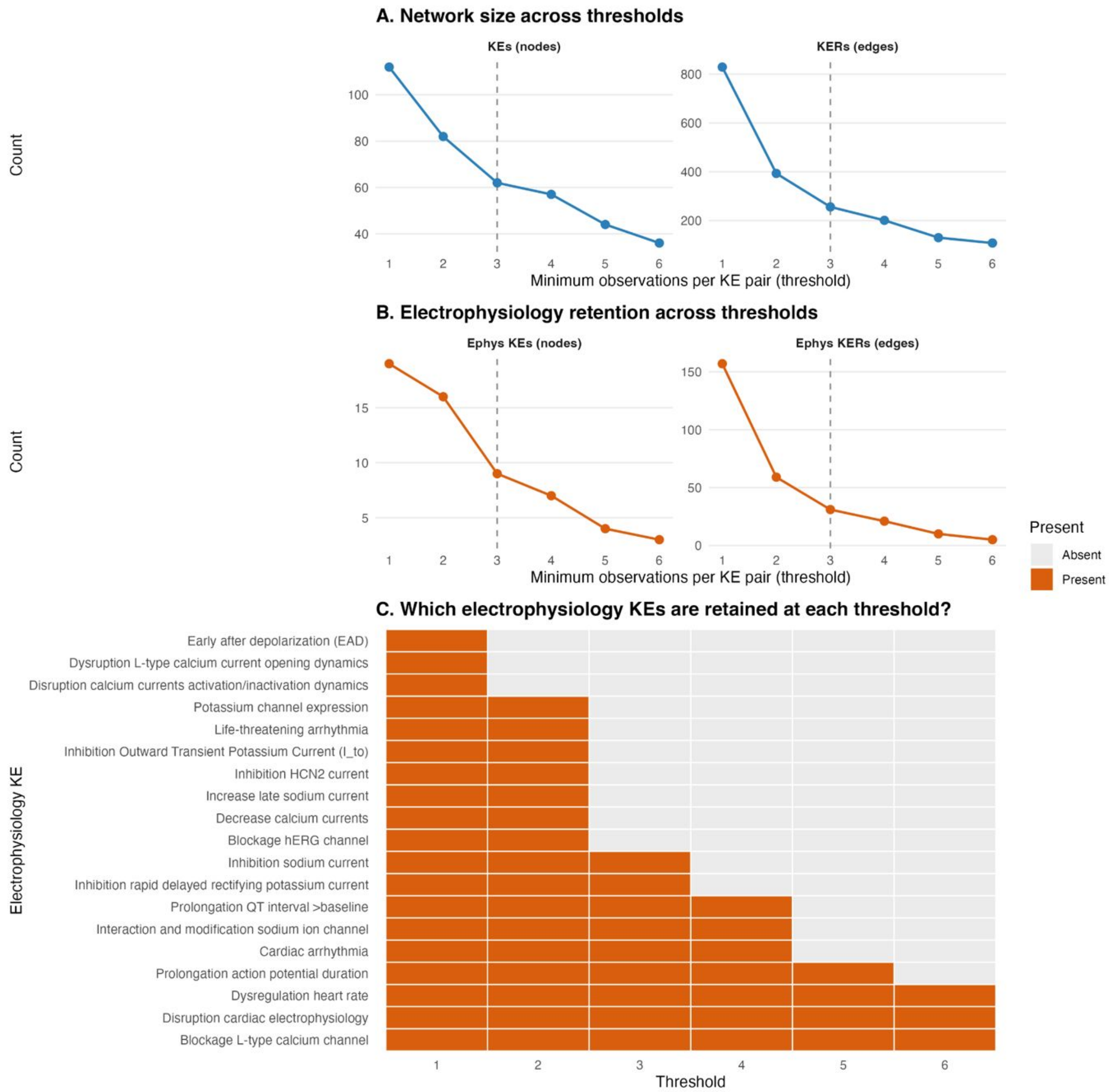

Figure S9: Threshold sensitivity analysis with electrophysiology tracking.
Networks were constructed at minimum observation thresholds ranging from ≥1 to ≥6 per KE–KE pair. (A) Total network size (KEs and KERs) across thresholds; dashed line indicates the threshold used in the main analysis (≥3). (B) Electrophysiology-related KE and KER retention across thresholds. (C) Presence/absence map showing which electrophysiology KEs are retained at each threshold (grey = absent; colored = present).

1. Bilgin, E. *AOP 433: hERG channel blockade leading to sudden cardiac death*. 2023 15th December 2025]; Available from: https://aopwiki.org/aops/433.
2. Margiotta-Casaluci, L. *AOP 261: L-type calcium channel blockade leading to heart failure via decrease in cardiac contractility*. 2023 15th December 2025]; Available from: https://aopwiki.org/aops/261.
3. Margiotta-Casaluci, L.D., H.; Moreira, I.; Winter, M. J.; Prior, H. *Development of an Adverse Outcome Pathway for cardiotoxicity mediated by the blockade of L-type calcium channels*. 2018 15th December 2025]; Available from: https://nc3rs.org.uk/sites/default/files/documents/Posters/SPS%20meeting%202018%20-%20AOP%20poster%20-%20Margiotta-Casaluci.pdf.

4. Ogienko, A.A., et al., *Drosophila as a Model Organism to Study Basic Mechanisms of Longevity.* Int J Mol Sci, 2022. **23**(19).
